# Enhanced sialic acid engagement at physiological temperatures by reovirus σ1 mutants facilitates infection of breast cancer cells with low levels of high-affinity receptors

**DOI:** 10.1128/jvi.00074-26

**Published:** 2026-03-12

**Authors:** Dirk Taal, Spencer Luong, Maia Walker, Heather E. Eaton, Paris I. Brown, Tim Footz, Maya Shmulevitz

**Affiliations:** 1 Department of Medical Microbiology and Immunology, Li Ka Shing Institute of Virology, University of Alberta 839763, Edmonton, Alberta, Canada; University of Michigan Medical School, Ann Arbor, Michigan, USA

**Keywords:** receptor engagement, oncolytic virus, Mammalian orthoreovirus (reovirus)

## Abstract

Reovirus serotype 3 (T3D^PL^) serves as a safe, tractable model for studying fundamental virus-host interactions and is also under investigation as an oncolytic therapy. Many viruses, including reoviruses, use sialic acids as attachment factors in addition to protein receptors; however, the relative contributions of these interactions, particularly at physiological temperatures, remain poorly defined. T3D^PL^ engages sialic acids and JAM-A proteins through the tail and head domains of the σ1 cell attachment protein, respectively. In this study, murine E0771 breast cancer cells were found to resist reovirus infection due to a deficiency in high-affinity receptors. T3D^PL^ bound to E0771 cells at 4°C but not 37°C, revealing that wild-type sialic acid interactions are thermally unstable at physiological temperatures. Exogenous JAM-A expression restored stable binding and infection at 37°C. Notably, engineered reovirus variants lacking the σ1 head domain but possessing either an RGD motif or specific mutations in the sialic acid-binding domain overcame the restriction and could bind and infect E0771 cells efficiently at 37°C. Through neuraminidase treatment and sialic acid-deficient and dependent cell models, enhanced interactions by sialic acid-binding domain mutations were empirically confirmed to be mediated by sialic acid. Serial passaging of σ1-truncated virus rapidly selected for mutations that strengthened sialic acid-binding at 37°C. Together, these findings demonstrate that high-affinity receptor requirements can be bypassed by enhancing interactions at physiological temperatures with sialic acids or through RGD interactions. This work provides insight into how viruses adapt to receptor scarcity and offers a strategy to overcome receptor heterogeneity in oncolytic virotherapy.

**IMPORTANCE:**

Reoviruses are promising oncolytic agents, yet clinical efficacy can be hindered by heterogeneous receptor expression in tumors. This study demonstrates that reovirus can bypass high-affinity receptor requirements by optimizing sialic acid interactions or incorporating RGD motifs. Crucially, the data reveal that wild-type reovirus attachment to sialic acids is thermally unstable at physiological temperature (37°C), a restriction masked by traditional 4°C assays. Specific mutations were found to stabilize these interactions at 37°C, providing a mechanistic basis for viral adaptation to receptor-deficient environments. These findings establish new experimental approaches to study attachment at 37°C, which can be applied broadly to discover unanticipated mutational effects on viral entry. Ultimately, evaluating virus-cell attachment under physiological conditions is essential for accurately predicting viral tropism and facilitates the design of next-generation oncolytic therapies better equipped to overcome receptor scarcity and thermal barriers in complex tumor environments.

## INTRODUCTION

Engineered or naturally occurring oncolytic viruses (OVs) offer a dual therapeutic mechanism against cancer: they selectively replicate within and directly lyse transformed cells, while simultaneously triggering an anti-tumor immune response that targets residual disease. Understanding the factors that determine OV success or failure is key to advancing both virotherapy and fundamental cancer biology. Specifically, investigating why an OV infects some but not all tumor cells within a single cancer type may reveal critical insights into the functional diversity of cells and the underlying mechanisms of virus-host interactions that drive viral tropism.

Mammalian orthoreovirus (reovirus), a naturally occurring, non-pathogenic enteric virus, is a candidate OV. The unmodified serotype 3 Dearing PL-lab strain (T3D^PL^) is well tolerated in Phase II/III clinical trials against various malignancies, including breast cancer (1–10). However, the therapeutic efficacy of T3D^PL^ remains limited, with response rates often underwhelming (10). For instance, combination trials in breast cancer patients reported an extension in median overall survival but failed to improve response rate or progression-free survival (10). Given the ongoing focus on breast cancer in T3D^PL^ clinical trials, understanding the mechanisms by which some breast tumors resist reovirus infection and developing strategies to overcome these restrictions is essential. Multiple host and environmental factors have been identified that restrict T3D^PL^ activity in breast tumors, including virus-inactivating zinc-dependent metalloproteases (11), insufficient activation of host signaling pathways, including p38 MAPK and RAS downstream pathways (12–14), and inadequate disarmament of antiviral responses (15).

Whether receptor availability fundamentally restricts T3D^PL^ infection in some breast tumor cells remains poorly characterized. Reovirus attachment to cells begins with low-affinity engagement of sialic acids on the cell surface through the tail domain of the σ1 attachment protein, followed by high-affinity attachment to the Junctional Adhesion Molecule-A (JAM-A) receptor via the σ1 globular head domain (16–20). The trimeric σ1 attachment protein of T3D^PL^ consists of three main domains: an N-terminal “tail,” a central “body,” and a C-terminal “head,” linked by flexible connectors that confer conformational flexibility (21). The N-terminal tail forms an α-helical coiled-coil stabilized by repeating heptads and interhelical interactions (22), anchoring the trimer to the pentameric λ2 turret at each vertex of the viral capsid via key N-terminal residues (23). The central body domain contains β-sheet structures and a carbohydrate-binding pocket that mediates low-affinity interactions with sialylated glycans, including α-linked sialic acids (23). The C-terminal head domain (residues 310–455) adopts a β-barrel fold with surface-exposed loops that mediate high-affinity binding to JAM-A, triggering downstream capsid rearrangements required for entry (24–26). Receptor binding primes T3D^PL^ internalization through receptor-mediated, clathrin-dependent endocytosis. The critical role of cell surface receptors in oncolytic viral tropism is illustrated by restricted adenovirus activity in breast cancers with low CAR expression (27) and by limited reovirus infection in JAM-A-deficient glioblastoma cells (17). Although JAM-A is frequently upregulated in breast tumors (27–32), its expression is highly heterogeneous. It remains unclear whether this variability constitutes a primary barrier to T3D^PL^ infection.

The current study identifies murine E0771 breast cancer cells as a model of resistance to T3D^PL^ oncolysis *in vitro*, due to a deficiency in high-affinity receptor. Susceptibility is restored through exogenous expression of the JAM-A high-affinity receptor or by utilizing engineered reovirus variants that bypass the requirements for this receptor. These variants, which lack the JAM-A-binding σ1 head domain, utilize either an arginine-glycine-aspartic acid (RGD) motif engineered to engage β1 integrins or specific mutations that enhance sialic acid-dependent attachment. Crucially, while wild-type (WT) reovirus-sialic acid interactions are found to be highly temperature dependent, mediating binding at 4°C but thermally unstable at physiological temperature (37°C), mutations in the sialic acid-binding domain significantly stabilize interactions at 37°C. Furthermore, serial adaptation experiments found that the loss of the JAM-A-binding domain imposes immediate selection pressure, driving the rapid emergence of these temperature-stabilizing mutations. Collectively, these results suggest that JAM-A heterogeneity can create barriers to T3D^PL^ efficacy in breast cancer and illustrate the broader evolutionary mechanism whereby viruses can adapt to receptor scarcity by optimizing low-affinity interactions to withstand physiological thermal stress.

## RESULTS

### E0771 cells represent a poorly susceptible breast cancer target for oncolytic reovirus infection

To date, analyses of T3D^PL^ in murine breast cancer models have focused primarily on xenograft systems, which fail to recapitulate the roles of immune cells in both clearing the virus and aiding the host in eliminating tumor cells (13, 33, 34). To better understand the limitations of T3D^PL^-mediated oncolysis in breast cancer, several syngeneic, immunocompetent mouse models are needed to capture both the direct tumor cell killing and the anti-tumor immunotherapeutic mechanisms of T3D^PL^. Ideally, if such models differ in their responses to T3D^PL^ therapy, they could help elucidate the factors limiting the direct and/or immunotherapeutic activities of reovirus. Toward this goal, we previously demonstrated that TUBO cells, a murine breast cancer cell line derived from a spontaneous mammary carcinoma in BALB/c mice, are susceptible to T3D^PL^ and that a reovirus mutant (SV5) that spreads more efficiently in tumors exhibits enhanced oncolytic activity compared to T3D^PL^ in TUBO models *in vivo* (35). Apart from the TUBO model, there remains a paucity of syngeneic immunocompetent murine breast cancer models available for investigating the mechanisms underlying susceptibility to T3D^PL^.

EMT6 and E0771 cells are commonly used in syngeneic immunocompetent murine breast cancer models (36–38). Like TUBO cells, EMT6 cells were derived from BALB/c mice; however, whereas TUBO cells are HER2/neu-positive, EMT6 cells represent a highly metastatic triple-negative breast cancer (TNBC) cell line. In contrast, E0771 cells, established from a spontaneous mammary carcinoma in C57BL/6 mice, are moderately metastatic and originate from a distinct genetic background. Although E0771 cells are sometimes classified as TNBC, there is evidence to suggest that they express estrogen receptor alpha (ERα^+^) (39–41). Given their utility in modeling distinct breast tumor subtypes in immunocompetent mice, the susceptibility of EMT6 and E0771 cells to T3D^PL^ was evaluated. The tumorigenic murine fibroblast cell line L929, which is highly permissive to T3D^PL^ infection, was included as a positive control. Cells were exposed to equal numbers of T3D^PL^ particles per cell. Because T3D^PL^ replication is typically complete within approximately 24 h in L929 and various other cell lines, including human breast cancer cells (42), the 18 h timepoint was selected to ensure robust viral antigen expression in productively infected cells. Productive infection was subsequently assessed by immunofluorescence microscopy and flow cytometry using a monoclonal antibody specific to σNS, a reovirus non-structural protein indicative of active viral replication.

**Fig 1:**
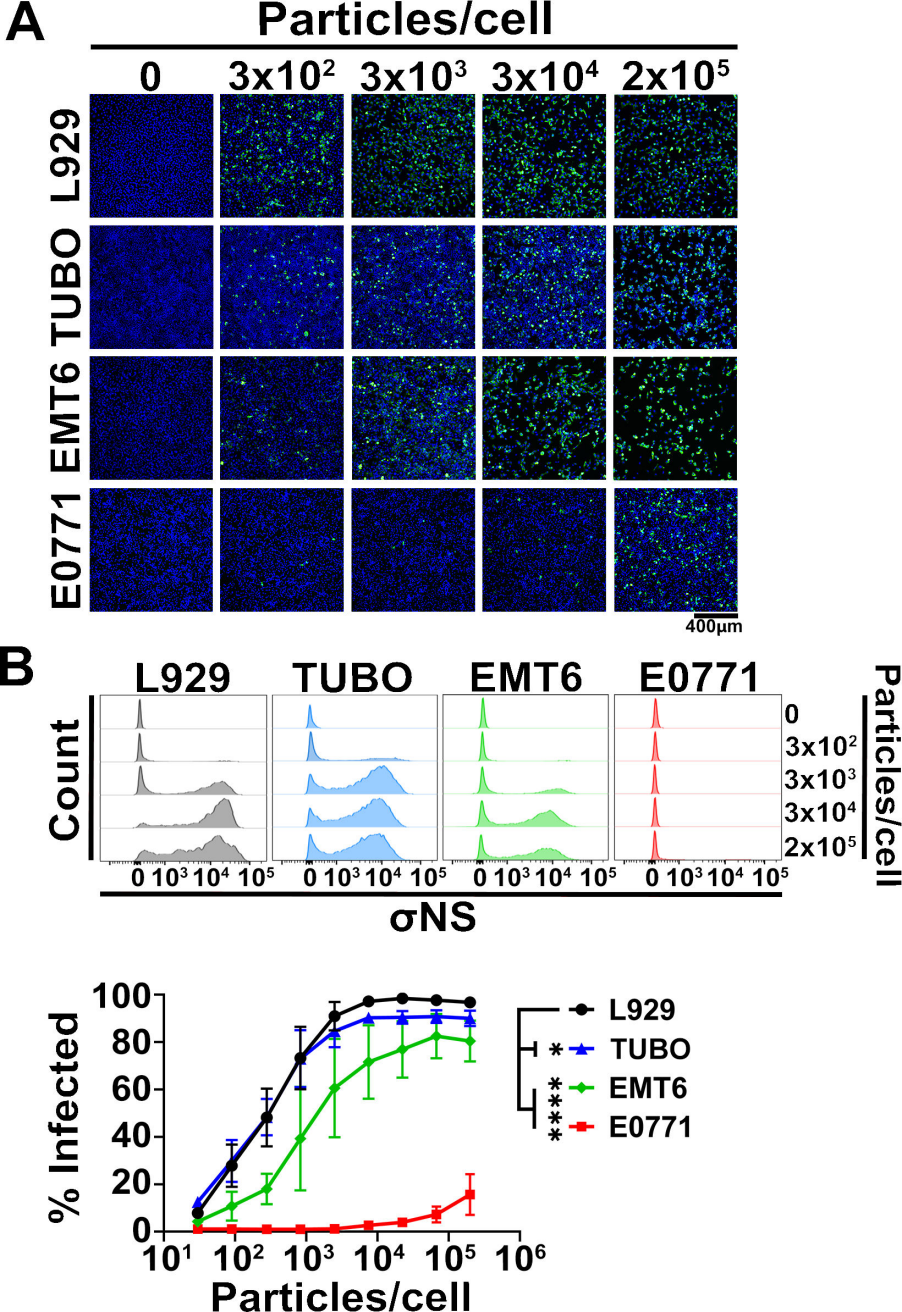
E0771 cells represent a poorly susceptible breast cancer target for oncolytic reovirus infection. L929, TUBO, EMT6, and E0771 cells were exposed to equivalent doses of T3D^PL^ particles per cell for 1 h at 37°C. Infection doses began at 6.8 × 10^5^ particles per cell, corresponding to a multiplicity of infection (MOI) of 3,000 on L929 cells and decreased by serial threefold dilutions. Unbound virus was removed by three PBS washes, and cells were incubated for 18 h at 37°C. (**A**) Cells were fixed with 4% PFA and analyzed for immunofluorescence microscopy following staining with anti-σNS antibodies (green) and DAPI (blue). Images were acquired at 10× magnification using an EVOS fluorescence microscope. (**B**) Top: Representative flow cytometric analysis of fixed cells immunostained with anti-σNS antibodies. Bottom: Percentage of productively infected (σNS-positive) cells plotted as a function of input particle number. Data represent mean ± SD (*n* = 3). Curves were fit using a four-parameter logistic (4PL) model, and statistical significance was assessed using an extra sum-of-squares F test in GraphPad Prism v10.4 (ns = *P* > 0.05; *P < 0.05; **P < 0.005; ***P < 0.001; and ****P < 0.0001).

Immunofluorescence analysis revealed a markedly lower number of σNS-positive cells in the E0771 cell line compared to L929, TUBO, and EMT6 cells when exposed to the same number of T3D^PL^ particles per cell, indicating that E0771 cells are substantially less susceptible to T3D^PL^ infection (Fig. 1A). Flow cytometric analysis indicated that both L929 and TUBO cells reached maximal percentage of infected cells (~95%) at 7.5 × 10^3^ particles per cell (Fig. 1B). The number of particles per cell required to infect 50% of cells (ID_50_) was calculated to be 282 and 289 for L929 and TUBO cells, respectively (Table 1). EMT6 cells required 6.8 × 10^4^ particles per cell to reach maximal infection (~80%), with an ID_50_ of 2.1 × 10^4^ particles per cell, representing a 7.5-fold decrease in susceptibility relative to L929 and TUBO cells. E0771 cells were very poorly infected, with only 18.8% of E0771 cells becoming productively infected even at 2.0 × 10^5^ particles per cell. The extrapolated ID_50_ for E0771 cells was 2.8 × 10^6^ particles per cell, corresponding to an approximately 10,000-fold decrease in susceptibility relative to L929 cells. Collectively, these data suggest that E0771 breast cancer cells are poorly susceptible to T3D^PL^ and represent a new model system for investigating the mechanisms that restrict efficient T3D^PL^ infection in certain breast cancers.

**TABLE 1:** Infectious dose 50% (ID_50_) and cell-surface receptor expression measured as median fluorescence intensity (MFI) across breast cancer cell lines

Cell line	Infectious dose 50% (ID_50_)^*a*^	Junctional adhesion molecule-A (MFI)	β1 integrin (MFI)	α2,6-linked sialic acid (MFI)
L929	282	49,563	12,860	10,727
E0771	2,781,969	2,093	15,288	17,873
EMT6	2,130	40,194	8,861	9,782
TUBO	289	ND^*b*^	ND	ND

^*a*^ Calculated using the curve equation taken from Fig. 1. Value for E0771 cells is an extrapolation.

^*b*^ ND, not determined.

### Reovirus attachment to E0771 cells is impaired at physiological temperature (37°C)

**Fig 2:**
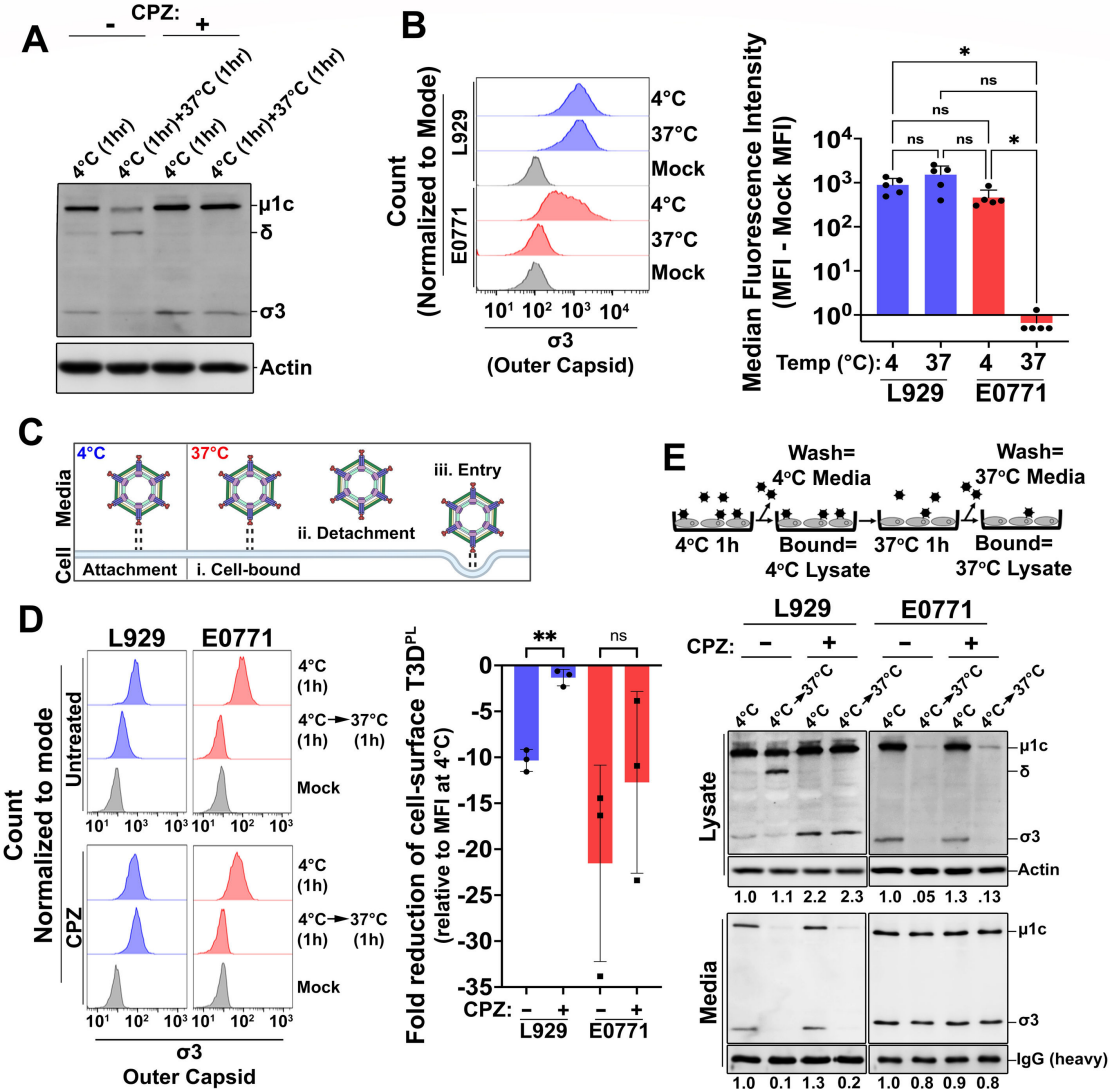
Reovirus attachment to E0771 cells is impaired at physiological temperature (37°C). (**A**) CPZ-treated or untreated L929 cells were exposed to 1.0 × 10^10^ T3D^PL^ particles for 1 h at 4°C. Unbound virions were removed by three PBS washes, followed by incubation at 37°C for 1 h. Cell lysates were collected at the indicated time points, resolved by SDS-PAGE, and analyzed by immunoblotting with anti-reovirus antibodies; β-actin served as a loading control. (**B**) L929 and E0771 cells were exposed to 1.0 × 10^10^ (L929) or 5.0 × 10^10^ (E0771) T3D^PL^ particles for 1 h at either 4°C (untreated) or 37°C (CPZ-treated). Following three PBS washes to remove unbound virions, cells were fixed with 4% paraformaldehyde (PFA), immunostained with σ3-specific monoclonal antibodies, and analyzed by flow cytometry. Representative histograms (left) and quantification of virion attachment by median fluorescence intensity (MFI; right) are shown. For visualization on a log scale, negative MFI values were reassigned a value of 0.5. Data represent mean ± SD (*n* = 5). (**C**) Schematic illustrating potential fates of T3D^PL^ virions bound at 4°C and subsequently warmed to 37°C for 1 h. Viruses graphics were created in BioRender (https://BioRender.com/k3z9gbu); figure assembly was performed in PowerPoint. (**D**) CPZ-treated or untreated L929 and E0771 cells were exposed to T3D^PL^ as in panel B for 1 h at 4°C. Following three PBS washes, cells were incubated at 37°C for 1 h and washed three additional times. Cells were collected at either 4°C or 37°C, and cell-associated virus was quantified by σ3 immunostaining and flow cytometry. Representative histograms (left) and quantification of virion detachment as fold change in MFI (right) are shown. Data represent mean ± SD (*n* = 3). (**E**) As depicted in panel C, the experiment in panel D was repeated in duplicate, and fractions remaining cell associated versus what was washed into the PBS washes were analyzed by western blotting with anti-reovirus antibodies; an irrelevant IgG served as a loading control. Band intensities were quantified using ImageLab software and normalized to T3D^PL^ binding to untreated cells at 4°C. Media and lysate samples were analyzed on separate blots; therefore, band intensities should be compared only within the same blot. Statistical significance was determined by the one-way ANOVA with Tukey’s multiple comparisons test in GraphPad Prism v10.4 (ns = *P* > 0.05; *P < 0.05; **P < 0.005; ***P < 0.001; and ****P < 0.0001).

The first step of productive infection by reovirus is attachment to cells. We therefore sought to determine whether poor cell attachment contributed to the low susceptibility of E0771 cells to T3D^PL^. To accurately replicate *in vivo* human body temperatures, attachment at physiological temperature (37°C) was of primary interest. However, immediately after binding, T3D^PL^ is internalized via receptor-mediated, clathrin-dependent endocytosis and partially disassembled in lysosomes (43–46). Specifically, the outer capsid protein σ3 is degraded, and the µ1c protein is cleaved to a smaller δ fragment (47, 48). To prevent internalization and focus on cell attachment, chlorpromazine (CPZ) was tested to inhibit clathrin-dependent endocytosis (45, 49). L929 cells were pre-treated with CPZ or left untreated, and virus adsorption was performed at 4°C for 1 h to allow attachment without entry in both conditions. Cells were then washed extensively to remove unbound virus and incubated at 37°C for 1 h in the presence or absence of CPZ. Western blot analysis showed that cell-associated virus was equal in CPZ-treated and untreated L929 cells following attachment at 4°C, as evidenced by comparable levels of undegraded outer capsid proteins σ3 and µ1c (Fig. 2A). In contrast, while σ3 degradation and µ1c cleavage to δ occurred in untreated cells after incubation at 37°C, both outer capsid proteins remained intact in CPZ-treated cells. These results indicate that CPZ effectively inhibited internalization.

Cell binding of T3D^PL^ was evaluated for E0771 versus L929 cells at both 4°C and 37°C for 1 h, using CPZ to inhibit internalization at 37°C. Fivefold more virus particles were used per cell for E0771 cells relative to L929 cells to increase the likelihood of detecting E0771-bound virions. After extensive washing to remove unbound virus, cells were fixed, subjected to immunostaining for the outer capsid protein σ3, and analyzed by flow cytometry. The median fluorescence intensity (MFI), used as a measure of the quantity of cell-associated whole virions, was similar for L929 cells at 4°C and 37°C (*P* = 0.49; Fig. 2B). In contrast, while the MFI of T3D^PL^ particles bound to E0771 cells was 461.1 ± 218.3 at 4°C, it decreased to 0.7 ± 0.4 at 37°C. This significantly reduced attachment of T3D^PL^ to E0771 cells at 37°C (*P* = 0.03) suggests that although reovirus can bind E0771 cells at 4°C, binding is minimal at physiological temperature (37°C). The approximately 1,000-fold lower binding at physiological temperature likely contributes substantially to the poor susceptibility of E0771 cells (Fig. 1).

To our knowledge, temperature-dependent attachment of reovirus has not previously been described. Attachment studies are typically conducted at a single temperature, most commonly at 4°C to preclude entry (16–20, 50). Given the importance of viral attachment at physiological temperatures, we conducted a comprehensive assessment of attachment versus detachment when switching from a bound state at 4°C to 37°C. L929 and E0771 cells were treated with CPZ prior to T3D^PL^ exposure at 4°C, followed by extensive washing and incubation at 37°C for 1 h. The experimental design allowed for the monitoring of three potential fates for virus particles upon the transition to 37°C: (i) remaining cell-bound, (ii) detaching into the media, or (iii) undergoing entry and uncoating (Fig. 2C). In L929 cells, transition to 37°C for 1 h led to a 10.3 ± 1.2-fold reduction in cell-surface T3D^PL^ virions for untreated L929 cells and a 1.3 ± 0.9-fold reduction for CPZ-treated L929 cells, suggesting that virions were predominantly internalized at 37°C (Fig. 2D). To directly monitor if virus particles are internalized or released at different temperatures, western blot analysis was used to determine if viruses remained associated with cells or released to the media, and whether cell-associated virus underwent uncoating (Fig. 2E). Cells were incubated with virus for 1 h at 4°C, followed by separation of cell-associated (cell lysate) versus unassociated (media) fractions. Having washed away the unassociated viruses, the cells were then transitioned to 37°C for 1 h and again separated into cell-associated (cell lysate) versus unassociated (media) fractions. In support of internalization rather than detachment in L929 cells, transition to 37°C in the absence of CPZ resulted in classical markers of reovirus uncoating, including degradation of σ3 and cleavage of µ1C to δ, and no virus particles became unassociated into the media following transition to 37°C (Fig. 2E). β-actin served as a loading control for cell lysates, while the media was artificially spiked with recombinant IgG to provide a loading control. Unlike L929 cells, transition to 37°C in E0771 cells led to a 21.5 ± 10.6-fold reduction in cell-surface virus particles, which remained statistically unchanged in CPZ-treated cells (12.7 ± 9.9-fold, *P* = 0.20; Fig. 2D). Virus particles detached into the media rather than being internalized after transition to 37°C (Fig. 2E). Taken together, these data suggest that T3D^PL^ can attach to E0771 cells at 4°C but not at 37°C, indicating that binding at 4°C is thermally unstable. These results reveal temperature-dependent differences in virus attachment to different cell types and emphasize the importance of attachment studies at physiological temperatures.

### E0771 cells have minimal expression of the high-affinity reovirus attachment receptor JAM-A

The reduced capacity of T3D^PL^ to attach to E0771 cells suggested a potential reduction in the expression of key cell surface receptors required for stable attachment and subsequent internalization of T3D^PL^. Attachment of T3D^PL^ is mediated by interactions between the fibrous σ1 attachment protein and various host cell surface receptors. The σ1 tail domain is capable of low-affinity interactions with sialylated glycans (51). The σ1 head domain mediates high-affinity, multivalent binding to JAM-A, a host cell adhesion protein (21, 24, 52). In addition to JAM-A, reovirus can also engage PirB and NgR1 as receptors in neurons and CNS-derived cells (53–59). Endocytosis is facilitated by subsequent engagement of the reovirus outer capsid protein λ2 with β1 integrins on the cell surface, via an RGD- and/or KGE-binding motif (60–62). Together, these coordinated interactions mediate the attachment and internalization of T3D^PL^ particles. A deficiency in high-affinity receptors, sialic acids, or β1 integrins could explain the failure of T3D^PL^ to attach to E0771 cells and establish a productive infection.

**Fig 3:**
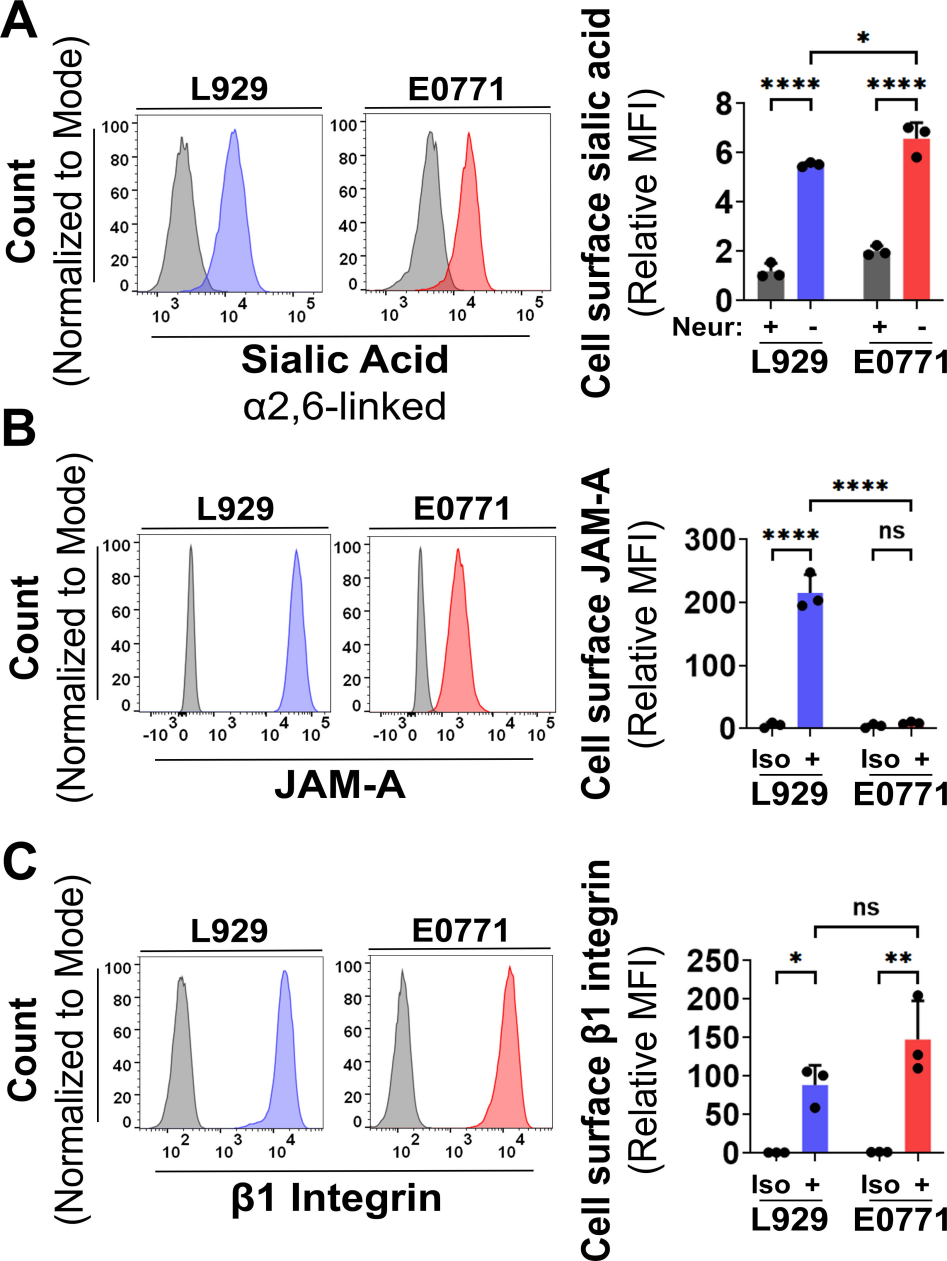
E0771 cells have reduced expression of the high-affinity reovirus attachment receptor JAM-A. E0771 and L929 cells were harvested using CellStripper, fixed with 4% paraformaldehyde (PFA), and analyzed by flow cytometry following immunostaining with monoclonal antibodies specific for SNA, JAM-A, or β1 integrin. Representative histograms (left) and corresponding quantification (right) show cell surface expression of (**A**) α2,6-linked sialic acids detected using fluorescently labeled SNA lectin in neuraminidase-treated (+) or untreated (−) cells, (**B**) JAM-A detected using murine JAM-A-specific antibodies (+) compared with isotype (Iso) controls, and (**C**) β1 integrin detected using β1 integrin-specific primary antibodies (+) compared with Iso controls. MFIs were normalized to the corresponding negative controls (neuraminidase-treated or isotype-stained cells) from a representative L929 experiment. Data represent mean ± SD (*n* = 3). Statistical significance was determined by the two-way ANOVA with Tukey’s multiple comparisons test in GraphPad Prism v10.4 (ns = *P* > 0.05; *P < 0.05; **P < 0.005; ***P < 0.001; and ****P < 0.0001).

To assess the abundance of sialic acids on the surface of E0771 cells, L929 cells were used for comparison, as they support T3D^PL^ attachment at physiological temperature (37°C). Fluorescently labeled plant lectins derived from *Sambucus nigra* agglutinin (SNA) were used to detect α2,6-linked sialic acids on untreated or neuraminidase-treated cells via flow cytometry (Fig. 3A). The MFI, used as a measure of sialic acid abundance, was significantly higher in E0771 cells than in L929 cells (*P* = 0.04). Treatment with neuraminidase reduced the MFI in both cell types, confirming the specificity of SNA (Fig. 3A). The high abundance of sialic acids on E0771 cells suggested that the failure of T3D^PL^ to attach at physiological temperature (37°C) was not due to a sialic acid deficiency.

Among the high-affinity receptors, JAM-A was selected for initial analysis due to its more widespread expression and prevalence in breast cancer cells compared to PirB and NgR1. The expression of JAM-A and β1 integrin on the surface of E0771 and L929 cells was evaluated using indirect antibody staining and flow cytometry. E0771 cells exhibited significantly lower levels of JAM-A compared to L929 cells (Fig. 3B), suggesting a deficiency in cell surface JAM-A (*P* < 0.0001). In contrast, there were no significant differences in the abundance of β1 integrins on the surface of E0771 cells compared to L929 cells (*P* = 0.12), indicating that variation in β1 integrin availability is unlikely to account for the reduced susceptibility of E0771 cells to T3D^PL^ (Fig. 3C). Altogether, these results suggest that while E0771 cells possess both sialic acids and β1 integrins, they are deficient in JAM-A.

### Exogenous JAM-A expression restores T3D^PL^ attachment to E0771 cells, overcoming limitations caused by high-affinity receptor deficiency

To determine whether the deficiency of high-affinity receptors directly accounts for the reduced attachment of T3D^PL^ to E0771 cells at physiological temperature (37°C), a human JAM-A (hJAM-A) coding sequence was cloned into the pBabe retroviral vector and transduced into E0771 cells. This approach generated a derivative cell line (E0771+JAM) stably expressing exogenous hJAM-A in an otherwise wild-type E0771 background. An empty vector was transduced into E0771 cells (E0771-JAM) as a control to account for any effects of transduction or selection, thereby isolating the specific contribution of JAM-A to T3D^PL^ interactions. Non-transduced parental E0771 cells (E0771-WT) were used to control for any transduction-associated artifacts. Human JAM-A was chosen for transgenic expression because T3D^PL^ engages both human and murine JAM-A (mJAM-A) with comparable efficiency, but hJAM permitted specific detection of the transgene product on murine E0771 cells using human JAM-A-specific antibodies (52).

**Fig 4:**
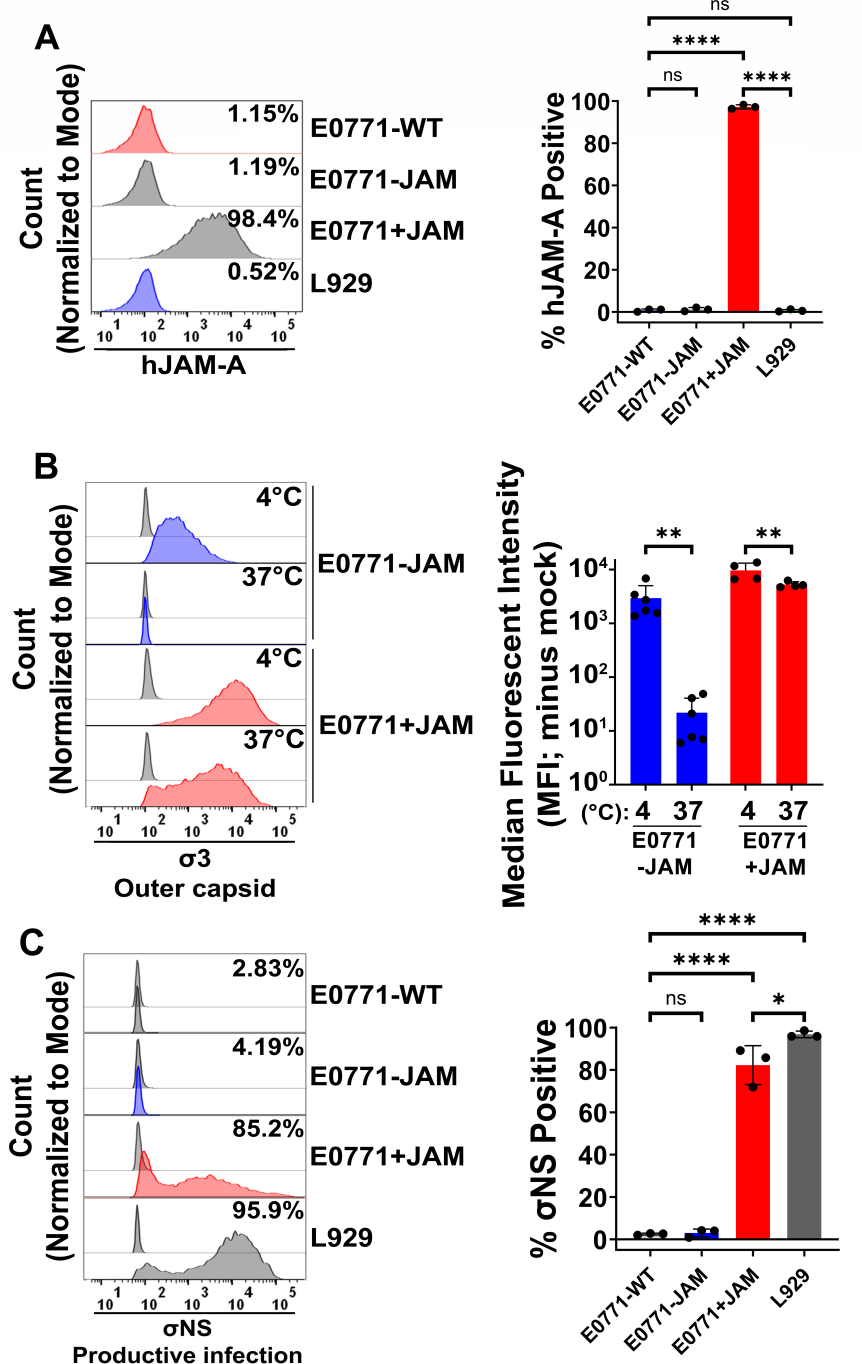
Exogenous expression of hJAM-A in E0771 cells is sufficient to restore infectivity of T3D^PL^. E0771 cells were transduced with a retroviral vector encoding hJAM-A on the pBabe backbone or with an empty pBabe vector and selected with puromycin to generate E0771+JAM and E0771-JAM cells, respectively. (**A**) E0771-WT (non-transduced), E0771-JAM, E0771+JAM, and L929 cells were fixed with 4% paraformaldehyde (PFA), immunostained for hJAM-A under non-permeabilizing conditions, and analyzed by flow cytometry. Representative histograms (left) show relative MFI, and quantification of surface JAM-A expression is graphed as the percentage of hJAM-A-positive cells (right). (**B**) E0771-JAM and E0771+JAM cells were exposed to equivalent T3D^PL^ particles for 1 h at either 4°C (untreated) or 37°C (CPZ-treated). After three PBS washes to remove unbound virions, cells were fixed with 4% PFA, immunostained with σ3-specific monoclonal antibodies, and analyzed by flow cytometry. Representative histogram (left) and quantification of virion attachment by MFI (right) are shown. For visualization on a log scale, negative MFI values were reassigned a value of 0.5. (**C**) E0771-WT, E0771-JAM, E0771+JAM, and L929 cells were incubated with or without T3D^PL^ at a multiplicity of infection (MOI) = 3 (relative to L929 titers) for 18 h at 37°C. Cells were fixed with 4% PFA, permeabilized, immunostained for σNS, and analyzed by flow cytometry. Representative histograms (left) and quantification of productively infected (σNS^+^) cells as a percentage of the total population (right) are shown. Data represent mean ± SD (*n* = 3). Statistical significance was determined by the one-way ANOVA with Tukey’s multiple comparisons test in GraphPad Prism v10.4 (ns = *P* > 0.05; *P < 0.05; **P < 0.005; ***P < 0.001; and ****P < 0.0001).

Successful transduction and surface expression of hJAM-A were verified by immunostaining E0771-WT, E0771−JAM, and E0771+JAM cells under non-permeabilizing conditions, followed by flow cytometric analysis (Fig. 4A). The specificity of the hJAM-A antibody was first validated using murine L929 cells, which exhibited minimal background signal, with only 0.7% ± 0.5% of cells positive for hJAM-A. Consistent with this baseline, E0771-WT and E0771-JAM cells showed low and statistically insignificant levels of hJAM-A-positive staining, averaging 0.8% ± 0.6% and 1.2% ± 0.9%, respectively (*P* = 0.91). In contrast, E0771+JAM cells demonstrated robust surface expression of hJAM-A, with an average of 97.3% ± 1.0% of the population staining positive.

To assess the impact of JAM-A expression on viral attachment, E0771-JAM and E0771+JAM cells were either untreated or pre-treated with CPZ prior to T3D^PL^ exposure for 1 h at 4°C or 37°C (Fig. 4B). MFI was used as a quantitative measure of T3D^PL^ attachment to the cell surface. At 4°C, E0771-JAM cells supported detectable T3D^PL^ binding (2,945 ± 1,895); however, attachment was nearly abolished at 37°C (21.9 ± 17.2, *P* = 0.004), consistent with previous observations in JAM-A-deficient E0771-WT cells. In contrast, E0771+JAM cells displayed markedly enhanced T3D^PL^ binding at both 4°C (9,677 ± 3,015) and 37°C (5,277 ± 586). The significant increase (*P* < 0.0001) in viral binding at physiological temperature strongly supports that deficiency of a high-affinity receptor such as JAM-A is an important factor limiting T3D^PL^ attachment in wild-type E0771 cells.

Although exogenous hJAM-A expression restored T3D^PL^ attachment at 37°C, it remained possible that downstream intracellular factors might independently restrict productive infection. E0771-WT, E0771-JAM, and E0771+JAM cells were infected with an equivalent multiplicity of infection (MOI) of T3D^PL^ and incubated for 18 h at 37°C prior to immunostaining for the non-structural protein σNS, followed by flow cytometric analysis (Fig. 4C). As a reference, L929 cells, which are highly permissive to T3D^PL^, showed 96.8% ± 1.6% σNS-positive cells. In comparison, E0771-WT and E0771-JAM cells displayed minimal infection, with 2.2% ± 0.4% and 3.1% ± 1.8% σNS-positive cells, respectively. Notably, E0771+JAM cells showed robust infection, with 82.3% ± 9.2% of the population positive for σNS, indicating that exogenous expression of hJAM-A is sufficient to support productive T3D^PL^ infection in this otherwise non-susceptible cell line (*P* < 0.0001). Accordingly, low attachment due to low levels of high-affinity receptor is the major cause of low susceptibility, rather than post-attachment limitations.

Collectively, these findings demonstrate that JAM-A expression is sufficient to restore T3D^PL^ attachment and replication in JAM-A-deficient E0771-WT cells. Moreover, since reovirus did not attach to E0771 cells until JAM-A was added, the results suggest that other known high-affinity receptors, such as PirB and NgR1, are likely absent or non-functional in E0771 cells, as they failed to compensate for the lack of JAM-A. Overall, the poor susceptibility of E0771 cells to T3D^PL^ can be largely attributed to the absence of a high-affinity receptor.

### Reovirus σ1-truncation variants productively infect JAM-A-deficient E0771 cells at physiological temperature (37°C)

**Fig 5:**
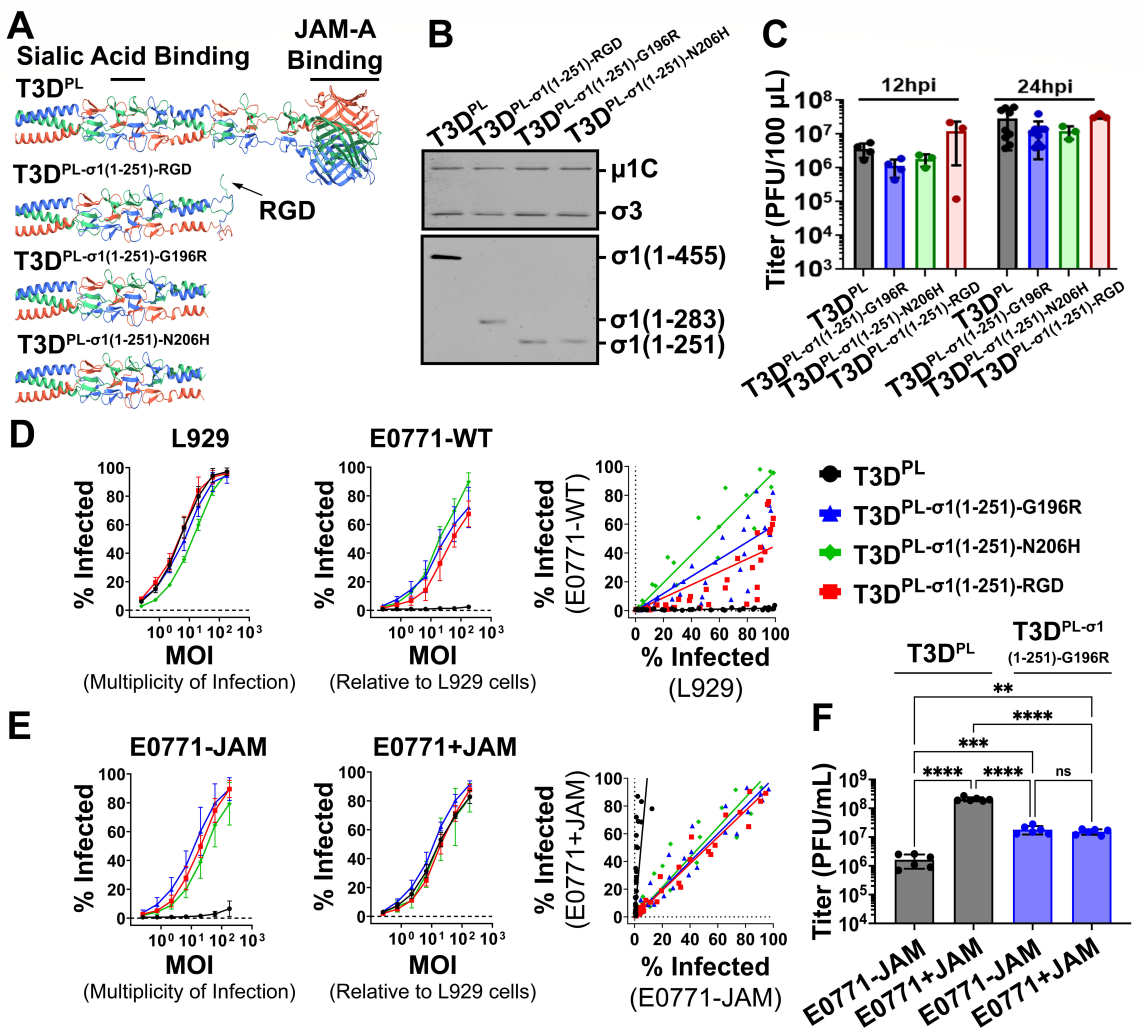
Reovirus σ1-truncation variants productively infect JAM-A-deficient E0771 cells at physiological temperature (37°C). (**A**) Ribbon diagrams of four σ1 constructs assessed for their capacity to attach to JAM-A-deficient E0771 cells. Individual monomers are colored red, blue, or green with the JAM-A and sialic acid-binding domains labeled. Top: Full-length σ1 (1–455; PDB: 6GAP, 3S6X). Second from top: Truncated σ1 (1–251) with the additional RGD motif, as predicted by AlphaFold. Second from bottom: Truncated σ1 (1–251) with G196R substitution mutation, as predicted by AlphaFold. Bottom: Truncated σ1 (1–251) with N206H substitution mutation, as predicted by AlphaFold. (**B**) Western blot analysis of pure virus, immunostained with polyclonal antibodies raised against reovirus (top blot) or toward the tail terminal domain of σ1 (bottom blot). (**C**) To establish replication proficiency, L929 cells were infected with T3D^PL^ versus indicated mutants at MOI = 5, and total lysates collected at 12 and 24 h post-infection were subjected to plaque titration on L929 cells. (D/E) L929 cells were infected with serial dilutions of matched MOIs of T3D^PL^, T3D^PL-σ1(1-251)-RGD^, T3D^PL-σ 1(1-251)-G196R^, or T3D^PL-σ1(1-251)-N206H^, based on titers determined in L929 cells. After 18 h at 37°C, cells were collected with CellStripper, fixed with 4% paraformaldehyde (PFA), and immunostained using a monoclonal antibody for reovirus σNS. Data represent mean ± SD (*n* = 3). (**D**) L929 and E0771-WT cells infected with T3D^PL^ and the σ1-truncation variants (left and middle). Comparison of percent infected between the indicated cell lines, with a line of best fit (right). (**E**) E0771-JAM and E0771+JAM cells infected with T3D^PL^ and the σ1-truncation variants (left and middle). Comparison of percent infected between the indicated cell lines, with a line of best fit (right). (**F**) E0771-JAM or E0771+JAM cells were infected with T3DPL or T3D^PL-σ 1(1-251)-G196R^ at an MOI of 10 (based on L929 titers). Total lysates collected at 24 h post-infection were subjected to plaque titration on L929 cells. Data represent mean ± SD (*n* = 6). Statistical significance was determined by the one-way ANOVA with Tukey’s multiple comparisons test in GraphPad Prism v10.4 (ns = *P* > 0.05; *P < 0.05; **P < 0.005; ***P < 0.001; and ****P < 0.0001).

Our lab has accumulated an extensive panel of reovirus mutants (11, 35, 42, 63–68). To determine whether any of these reovirus variants can productively infect E0771 cells, we screened our panel for their ability to infect these cells. Three variants were found capable of infecting E0771 cells: T3D^PL-σ1(1-251)-G196R^, T3D^PL-σ1(1-251)-N206H^, and T3D^PL-σ1(1-251)-RGD^. In all three variants, the JAM-A-binding head domain of σ1 was removed by addition of a stop codon at position 252 of σ1, leaving behind only the sialic acid-binding tail domain of σ1 (Fig. 5A). Variants T3D^PL-σ1(1-251)-G196R^ and T3D^PL-σ1(1-251)-N206H^ carry truncated σ1 proteins with a single amino acid substitution (G196R or N206H, respectively). T3D^PL-σ1(1-251)-RGD^ was engineered via site-directed mutagenesis to introduce an RGD motif at the C-terminal end of the otherwise unmutated, truncated σ1 protein. This motif mimics a known β1 integrin-binding sequence in the λ2 turret and was designed to promote β1 integrin engagement (62, 69, 70). The size of truncated σ1 on purified virus particles was confirmed for T3D^PL-σ1(1-251)-G196R^ and T3D^PL-σ1(1-251)-RGD^ by western blot analysis (Fig. 5B). Furthermore, in a single-step growth analysis (MOI = 5), there were no significant differences in the progeny titers produced by the variants versus T3D^PL^ at both 12 and 24 h post-infection in L929 cells, indicating that the variants do not have replication deficiencies following viral entry (Fig. 5C).

The ability of σ1-truncation variants versus wild-type reovirus particles to establish infection in L929 cells versus E0771 cells was then assessed. Cells were exposed to serial dilutions of each variant and T3D^PL^ for 18 h at 37°C, and productive infection was measured by flow cytometric analysis of non-structural protein σNS expression (Fig. 5D, left). All three σ1-truncation variants exhibited comparable infectivity profiles on JAM-A-sufficient L929 cells. Next, E0771-WT cells were exposed to T3D^PL^ or the σ1-truncation variants at 37°C at a MOI equivalent to that used for L929 cells (Fig. 5D, middle). While T3D^PL^ was largely unable to establish productive infection in JAM-A-deficient E0771-WT cells, all three σ1-truncation variants demonstrated a robust capacity to establish infection in E0771-WT cells (Fig. 5D, middle). When comparing the percentage of infected cells between the two cell lines, the σ1-truncation variants, but not T3D^PL^, exhibited a strong linear correlation (Fig. 5D, right), indicating that their infectivity is governed by a common, JAM-A-independent factor present in both L929 and E0771 cells. The σ1-truncation variants also infected E0771-JAM cells (Fig. 5E, left) and E0771+JAM cells (Fig. 5E, middle) with comparable efficiency. Consistent with the findings in E0771-WT, the correlation analysis in Fig. 5E (right) suggests that the variants utilize a consistent attachment strategy regardless of JAM-A status. In contrast, T3D^PL^ required JAM-A expression to infect E0771 cells. Together, these results suggest that the three σ1-truncation variants, T3D^PL-σ1(1-251)-G196R^, T3D^PL-σ1(1-251)-N206H^, and T3D^PL-σ1(1-251)-RGD^, can mediate infection via JAM-A-independent mechanisms.

To assess whether these differences in infection efficiency also translate into differences in infectious progeny production, E0771-JAM or E0771+JAM cells were infected with T3D^PL^ or T3D^PL-σ1(1-251)-G196R^ at an MOI of 10, normalized based on L929 titers, and total progeny virus was measured at 24 h post-infection by plaque assay (Fig. 5F). T3D^PL^ produced the highest progeny titers in JAM-A-expressing E0771 cells but exhibited markedly reduced replication in the absence of JAM-A. In contrast, T3D^PL-σ1(1-251)-G196R^ showed intermediate progeny production overall and supported enhanced replication in JAM-A-deficient cells relative to T3D^PL^, though it did not reach the peak titers achieved by T3D^PL^ in the presence of JAM-A. These patterns suggest that while the engineered variants provide a robust alternative entry pathway to bypass JAM-A, the native JAM-A-mediated entry route remains more efficient for maximal progeny production. Collectively, these results indicate that the variants were capable of infecting E0771 cells at physiological temperatures by utilizing alternative cell surface moieties.

### Reovirus σ1-truncation variants have improved attachment to JAM-A-deficient E0771 cells at physiological temperature (37°C)

**Fig 6:**
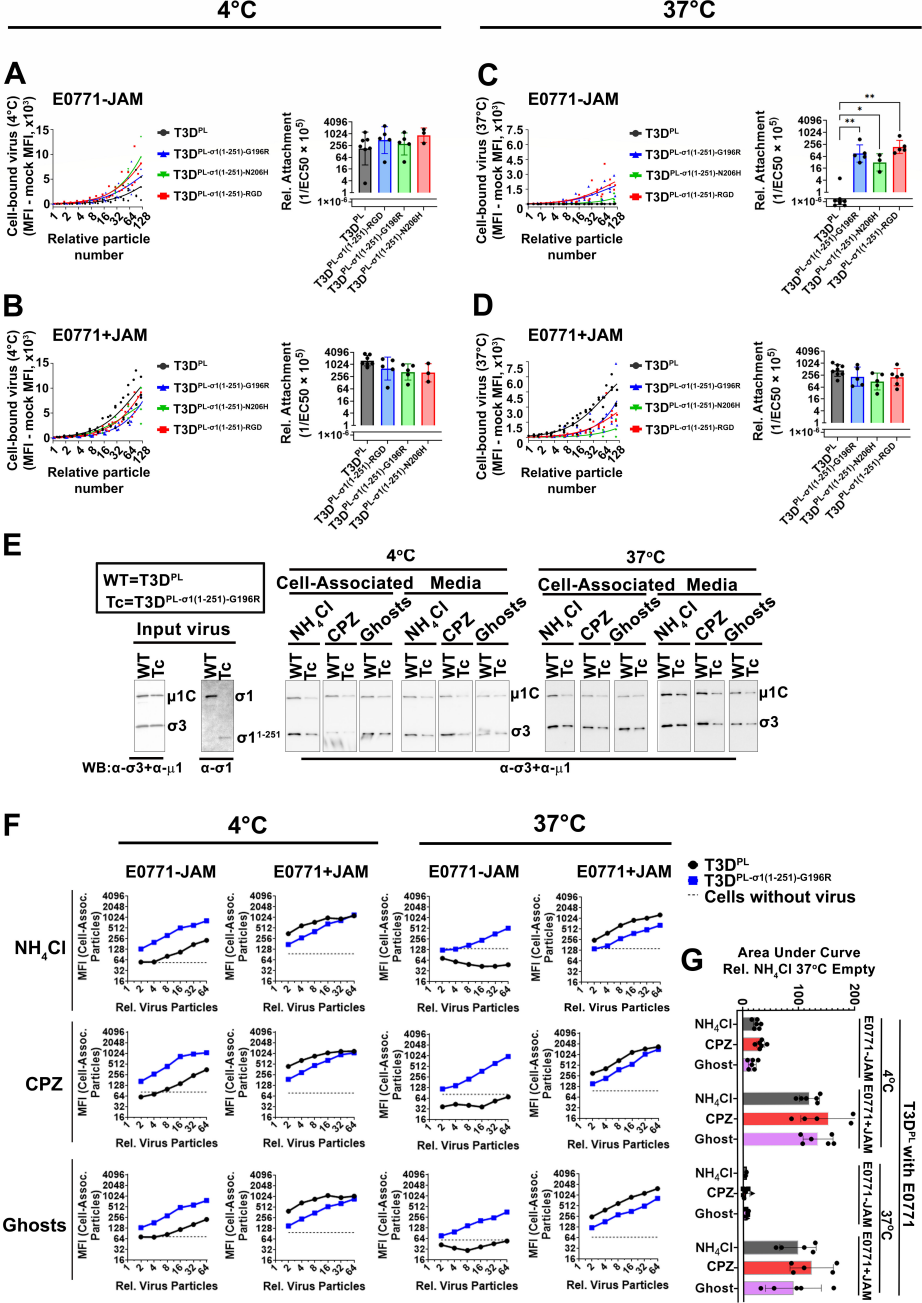
Reovirus σ1-truncation variants have improved attachment to JAM-A-deficient E0771 cells at physiological temperature (37°C). (**A–D**) Confluent monolayers of E0771-JAM cells (**A and C**) or E0771+JAM cells (**B and D**) were either untreated or pre-treated with CPZ and then exposed to a twofold serial dilution of T3D^PL^, T3D^PL-σ1(1-251)-RGD^, T3D^PL-σ1(1-251)-G196R^, or T3D^PL-σ1(1-251)-N206H^ virions starting at 5.7 × 10^10^ particles for 1 h at either 4°C (untreated cells; **A and B**) or 37°C (CPZ-treated cells; **C and D**). After three PBS washes to remove unbound virions, cells were fixed with 4% paraformaldehyde (PFA), immunostained with reovirus outer capsid protein (µ1C and σ3)-specific antibodies, and analyzed by flow cytometry. XY plots (left) show MFI representing virus attachment. Relative attachment (right) was calculated as the reciprocal of the EC50 times 10^5^ using an absolute EC50 model constrained to the global maximum MFI using GraphPad Prism v10.4. Significance was determined by one-way ANOVA on log-transformed data to account for log-normal distribution (*P < 0.05 and **P < 0.01). Data represent mean ± SD (*n* = 3–7). (**E**) To monitor the stability of outer capsid proteins µ1C and σ3 under different treatments, L929 cells were exposed to T3D^PL^ or T3D^PL-σ1(1-251)-G196R^ for 1 h at 4°C or 37°C (i) in the presence of NH_4_Cl during attachment, (ii) under CPZ pre-treatment for 1 h and during attachment, or (iii) after the L929 cells were first pre-fixed with 4% PFA (“Ghosts”). Cells were centrifuged, and the media (unbound virions) was collected. Cells were washed twice with PBS, and the final cell pellet (cell-associated virions) was collected. Media and lysates were subjected to western blot analysis using the indicated antibodies. Cell pellets and media were loaded on separate blots for each temperature, and virus particles were not equalized for this assay; therefore, the blots are intended to assess the prevention of reovirus uncoating rather than provide quantitative comparisons between conditions. (**F**) Quantitative assessment of attachment of serially diluted T3D^PL^ and T3D^PL-σ1(1-251)-G196R^ particles to E0771-JAM or E0771+JAM cells under three different treatment conditions: addition of NH_4_Cl during attachment (top row); CPZ pre-treatment for 1 h and during attachment (middle row), or pre-fixation of cells with 4% PFA (“Ghosts”) at 4°C or 37°C (bottom row), as indicated. Subsequent processing for flow cytometry was similar to panels A–D. (**G**) Results of five independent experiments similar to panel E using T3D^PL^ serial dilutions were analyzed by the two-way ANOVA with Tukey’s multiple comparisons test in GraphPad Prism v10.4 (ns = *P* > 0.05; *P < 0.05; **P < 0.005; ***P < 0.001; and ****P < 0.0001).

To directly test whether JAM-A-independent cell attachment underlies the enhanced infectivity of the σ1-truncation variants on E0771 cells, the binding capacity of the variants to JAM-A-deficient cells at 4°C and physiological temperature (37°C) was measured. Serial dilutions of virus particles, equalized between variants, were adsorbed onto E0771-JAM (empty vector control) and E0771+JAM (hJAM-A-expressing) cells. The cells were left untreated or treated with CPZ, incubated with virus at 4°C or 37°C for 1 h, and virus attachment to cells was quantified via flow cytometry. To compare binding efficiency across variants, dose-response curves were generated by plotting MFI against viral particle concentration. The EC50 (the number of particles required to achieve 50% of maximum binding) was determined for each replicate using non-linear regression. To ensure comparability across variants with different binding capacities, a global “Top” constraint was applied based on the global maximum MFI. Data are expressed as Relative Attachment (1/EC50 × 10^5^), such that higher values represent superior virus attachment. At 4°C on E0771-JAM cells, T3D^PL^ bound on average ~0.4-fold × less than and T3D^PL-σ1(1-251)-RGD^ and 0.8-fold × less than T3D^PL-σ1(1-251)-G196R^, T3D^PL-σ1(1-251)-N206H^, but differences were not statistically significant (Fig. 6A). At 4°C on E0771+JAM cells, T3D^PL^ bound on average ~1.8-fold × more than T3D^PL-σ1(1-251)-RGD^ and ~3.2-fold × more than T3D^PL-σ1(1-251)-G196R^ and T3D^PL-σ1(1-251)-N206H^, but differences were not statistically significant (Fig. 6B). However, at physiological temperature (37°C), T3D^PL^ exhibited minimal binding to E0771-JAM cells, while all three σ1-truncation variants showed significantly enhanced attachment (Fig. 6C). The inability of non-linear regression models to derive a confident EC50 for T3D^PL^ on E0771-JAM cells at 37°C further underscores the thermal instability of the wild-type interaction in the absence of a high-affinity receptor. Expression of JAM-A in E0771 cells relieved the temperature-dependent restriction, allowing T3D^PL^ attachment at physiological temperatures (Fig. 6D). Altogether, these results suggest that while attachment of the T3D^PL^ parent strain in the absence of a high-affinity receptor like JAM-A is highly temperature sensitive, the σ1-truncation variants exhibit a broad, more temperature-independent mechanism that bypasses this restriction at physiological temperatures.

Attachment is typically not measured at 37°C due to the confounding effects of internalization and onset of virus infection. While results with CPZ treatment suggested that physiological temperature is critical for differentiating the attachment capabilities of reovirus variants, it became essential to ensure that the observed effects were not due to unexpected indirect consequences of CPZ treatment. As an alternative approach, we treated cells with ammonium chloride (NH_4_Cl) during reovirus attachment. This treatment does not affect attachment or entry but prevents the uncoating of virions in late endosomes, thereby inhibiting viral exit from lysosomes and the onset of infection (12, 71, 72). Although NH_4_Cl does not distinguish between binding and endocytosis, it preserves fully intact particles with σ3 outer capsid proteins for continued detection by flow cytometry with σ3-specific antibodies and provides a CPZ-independent method for the earliest steps at 37°C. As another approach, we fixed cells with paraformaldehyde (PFA) to create “ghosts” incapable of cellular activities such as internalization, but merely to preserve presentation of cell proteins and carbohydrates on the cell surface; these ghosts were then used for virus attachment studies. The conditions were first tested to ensure that they prevented the uncoating of reovirus outer capsid proteins in lysosomes that occurs at 37°C during reovirus entry. To ensure lack of degradation of σ3 and cleavage of µ1C to δ that occurs during uncoating in untreated live L929 cells (Fig. 2A and E), the highly susceptible L929 cells were subjected to T3D^PL^ and T3D^PL-σ1(1-251)-G196R^ under each of the three conditions, and viruses associated with cells or retained in the media were subjected to non-quantitative western blot analysis (Fig. 6E). Under all conditions, reovirus outer capsid proteins σ3 and µ1C were stably maintained.

The three methods were then used to quantify attachment of T3D^PL^ and T3D^PL-σ1(1-251)-G196R^ to E0771 cells in the presence or absence of JAM-A. Both T3D^PL^ and T3D^PL-σ1(1-251)-G196R^ exhibited similar cell-association profiles in experiments using NH_4_Cl, CPZ, and ghost cells (Fig. 6F). For all three approaches, T3D^PL-σ1(1-251)-G196R^, but not T3D^PL^, attached to E0771 cells at 37°C. Because the ghost cell data, which precludes endocytosis, mirrors the NH_4_Cl and CPZ data, this strongly suggests that the association measured at 37°C is primarily driven by stable surface attachment. Area Under the Curve (AUC) was used to statistically compare the three methods using T3D^PL^ attachment to E0771 plus-or-minus JAM-A at 4°C and 37°C. AUC was selected over linear regression since it provided a more reliable metric for conditions with negligible attachment that sometimes could not derive an EC50 value (such T3D^PL^ on E0771-JAM 37°C), and also provided a direct linear relationship with attachment efficiency without the need for reciprocal transformations (i.e., 1/EC50). Note that to be able to use AUC, unlike previous experiments, we henceforth always used an identical relative number of particles between all independent experiments; this is because AUC requires identical x-axis parameters to accurately compare curves. There were no statistically significant differences between the three methods for measuring attachment at both 4°C and 37°C (Fig. 6G), supporting the use of all three approaches.

Together, these results demonstrate that the σ1-truncation variants can bind JAM-A-deficient E0771 cells at physiological temperature (37°C). This high-affinity, receptor-independent attachment likely explains their ability to establish productive infection and suggests that mutations in the sialic acid-binding domain or by introduction of an RGD domain facilitate alternative virus-cell engagement.

### Removal of the σ1 JAM-A attachment domain applies selection pressure that promotes mutations in the sialic acid-binding region

**Fig 7:**
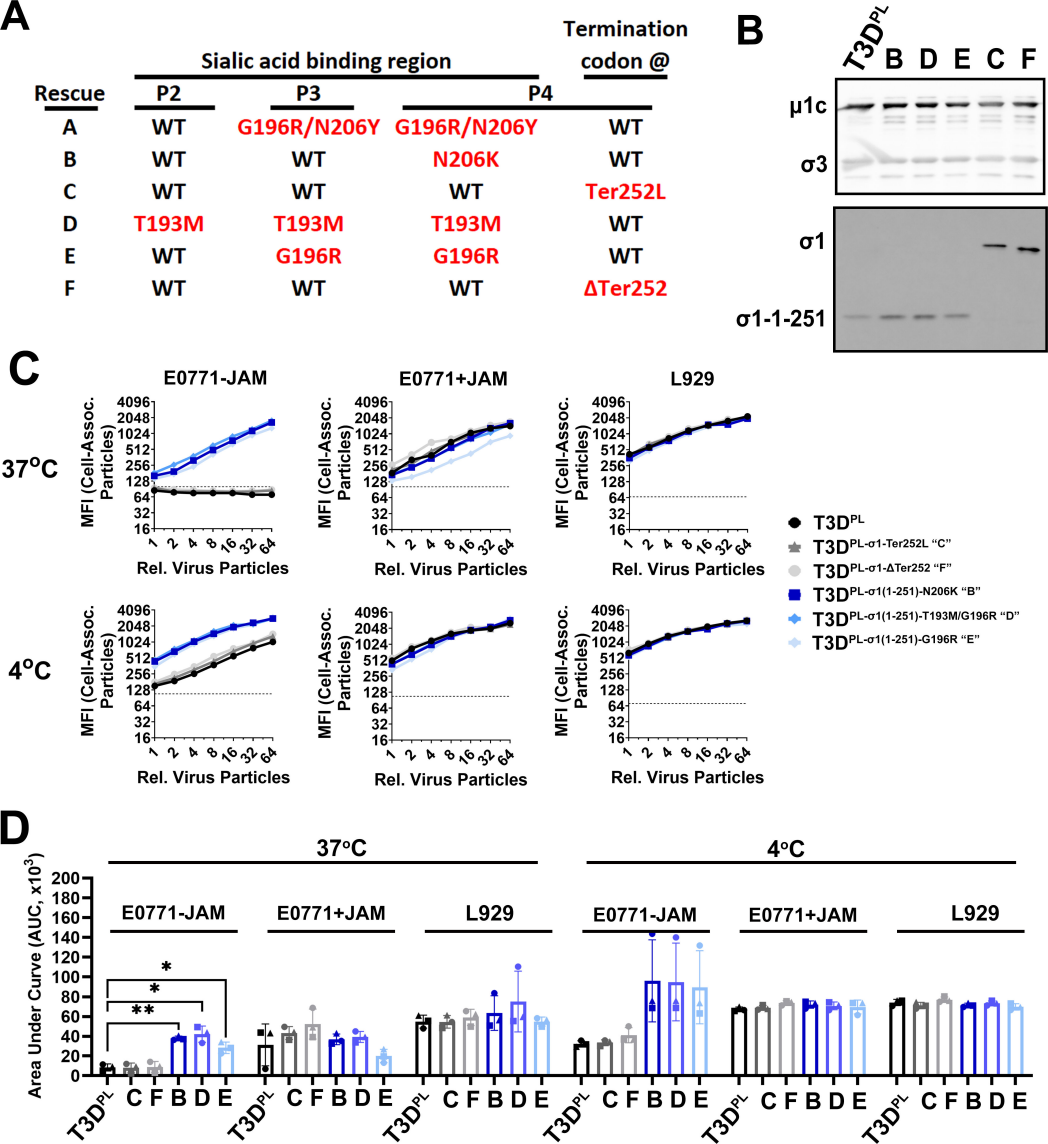
Removal of the σ1 JAM-A attachment domain applies selection pressure that promotes mutations in the sialic acid-binding region. (**A**) A stop codon was introduced at amino acid 252 of σ1, and viruses were generated using reverse genetics. Rescued viruses were sequenced after passages 2–4, as described in the text. The fate of mutations in the sialic acid-binding region or at the termination codon is shown for six independent rescues. (**B**) Western blot analysis of T3D^PL^ versus rescued variants “B–F” was performed with polyclonal anti-reovirus antibodies (top) or monoclonal anti-σ1 tail domain antibodies. Blot confirms that particles were effectively equalized for subsequent binding analysis and that outer capsid proteins µ1C and σ3 are intact and show the relative migration of truncated σ1 (variants B, D, and E), full-length σ1 (T3D^PL^ and variant C), or stop-codon-deleted σ1 (variant F). (**C**) Association of serially diluted equivalent particle numbers of the indicated viruses was measured on E0771-JAM-A, E0771+JAM-A, or L929 cells at 4°C or 37°C in the presence of NH_4_Cl. Flow cytometry was performed using outer capsid protein (µ1C and σ3)-specific antibodies. (**D**) AUC from three independent experiments shown in panel **C** was quantified. Statistical analysis was performed by the two-way ANOVA with Tukey’s multiple comparisons test in GraphPad Prism v10.4 (ns = *P* > 0.05; *P < 0.05; **P < 0.005; ***P < 0.001; and ****P < 0.0001).

Both T3D^PL-σ1(1-251)-G196R^ and T3D^PL-σ1(1-251)-N206H^ emerged spontaneously when we attempted to engineer a wild-type truncated σ1 by introducing a stop codon at position 252. The emergence of G196R and N206H mutations within the sialic acid-binding region of these σ1 truncation variants suggested that the absence of the σ1 JAM-A-binding head domain may drive the selection of mutations that promote attachment through JAM-A-independent mechanisms. To further investigate this hypothesis, we attempted again to obtain a wild-type truncated σ1 for use as a control while also determining whether mutations reproducibly arise when secondary receptors are unavailable. The reverse genetics plasmid encoding σ1 with a stop codon at position 252 was sequenced and verified to contain no additional mutations. Six independent reverse genetics reactions were conducted, propagated once on a 4.8 cm^2^ well of L929 cells (passage 1), twice more on 10 cm^2^ wells (passages 2 and 3), and finally scaled up to a 150 cm² dish (passage 4) to produce sufficient virus particles for subsequent attachment studies. By passage 4, all viruses incurred mutations. Specifically, four of the six viruses had mutations in the sialic acid-binding region (G196R/N206Y, N206K, T193M, or G196R; Fig. 7A) but retained the stop codon at position 252 and therefore produced truncated σ1 proteins by western blot analysis (Fig. 7B). Interestingly, the remaining two viruses (C and F) effectively “reversed” the truncation by eliminating the introduced stop codon, thereby restoring a full-length or near-full-length σ1 protein (Fig. 7A and B). Specifically, one mutated the stop codon to a leucine-coding codon and produced full-length σ1, while another deleted the three nucleotides of the stop codon and produced a σ1 that migrates slightly faster than full length σ1. The biological reversion to a JAM-A-binding phenotype further emphasizes that a wild-type truncated σ1 tail is insufficient for stable propagation. These results indicated that the removal of the σ1 head domain imposes selective pressure that favors the emergence of compensatory mutations in the sialic acid-binding domain. These results indicate that the removal of the $\sigma1$ head domain imposes severe selective pressure favoring the emergence of compensatory mutations. Despite extensive efforts, a wild-type truncated σ1 could not be isolated by passage 4, likely because it is incapable of the stable attachment required for viral rescue and propagation. Consequently, full-length σ1 served as the control for the wild-type sialic acid-binding sequence.

The attachment of newly rescued T3D^PL^ variants containing single codon mutations was evaluated on E0771 cells with and without hJAM-A exogenous expression, as well as on L929 cells. Both T3D^PL-σ1-Ter252L^ (virus “C”) and T3D^PL-σ1-ΔTer252^ (virus “F”) were similar to T3D^PL^, showing no attachment to JAM-A-deficient E0771 cells at physiological temperature (Fig. 7C and D). In contrast, the mutations N206K, T193M, and G196R displayed significantly enhanced JAM-A-independent attachment at 37°C. Collectively, the emergence of multiple distinct stabilizing mutations (T193, G196, and N206) across independent experiments suggests that the 37°C thermal barrier is a primary evolutionary driver for viral adaptation in receptor-limited environments. Altogether, these results strongly suggest that mutations in the sialic acid-binding domain or the addition of an RGD domain enhance JAM-A-independent attachment mechanisms in E0771 cells at physiological temperature.

### Attachment of reovirus variants to E0771 cells at physiological temperature is not caused by increased quantity of σ1 attachment proteins on virions

Reovirus is an icosahedral, non-enveloped virus with 12 vertices, each with the potential to hold a σ1 trimer. Larson et al. (73) discovered that reovirus particles can be resolved into 13 bands by agarose gel electrophoresis, with each band representing particles that possess 0–12 σ1 trimers (73). Different reovirus lab strains and variants show varying ranges of σ1 trimers, with T3D^PL^ having one of the highest averages per virion (42, 66, 67). Mutations that reduced the average number of σ1 trimers per virion, while maintaining sufficient trimers for attachment, were found to enhance the oncolytic potency of reovirus by promoting σ1 removal during entry and decreasing immediate attachment, thus promoting more successful onset of infection and further distance of dissemination, respectively (35, 63, 65). Subsequent studies identified additional mutations affecting the levels of σ1 trimers per virion (74–76).

**Fig 8:**
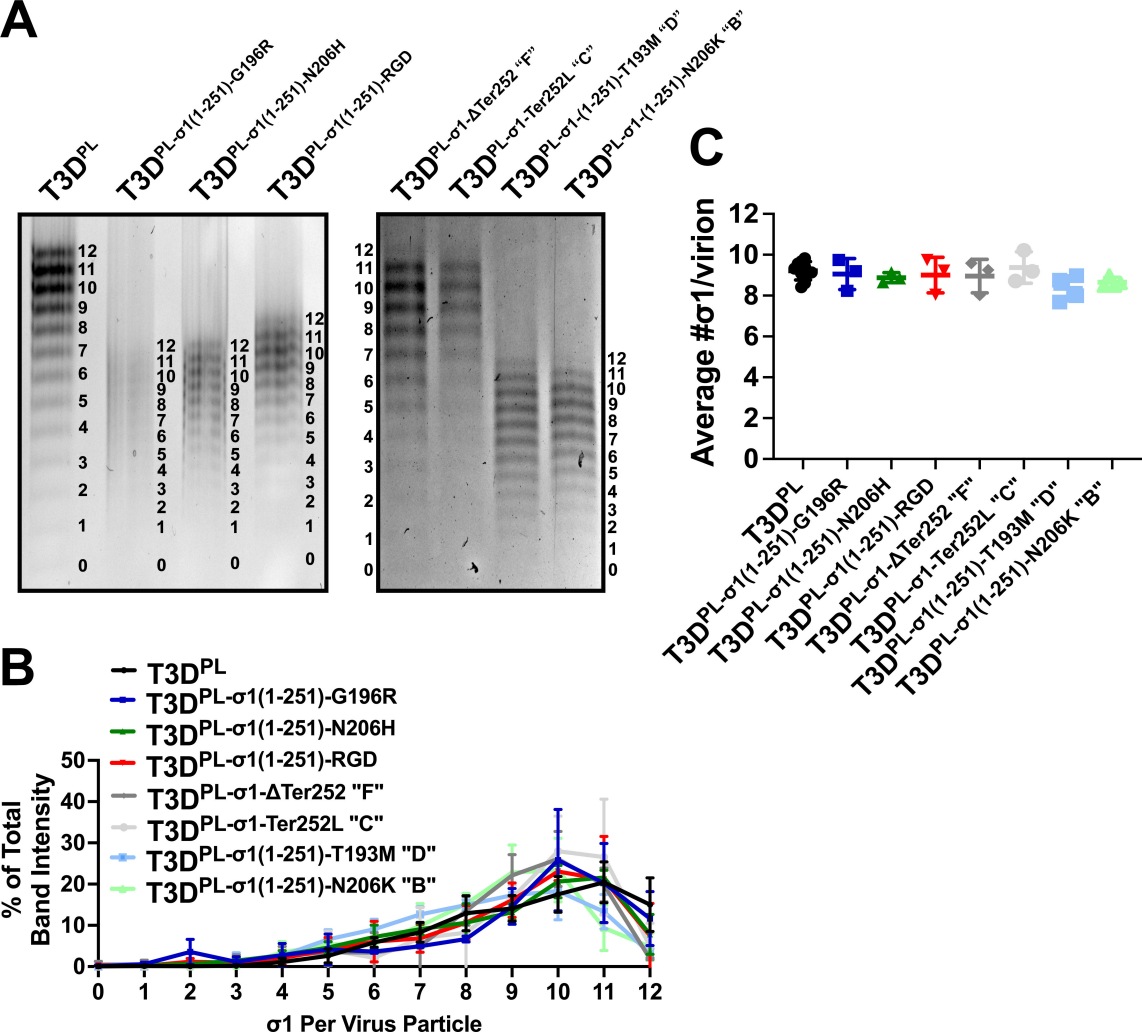
Increased attachment of reovirus variants is not due to higher levels of σ1 trimers per virion. (**A**) Agarose gel electrophoresis of reovirus particles showing distinct bands corresponding to 0–12 σ1 trimers. (**B**) Quantification of band intensities as a percentage of the total signal per virus particle. (**C**) Calculated average number of σ1 trimers per virion across different variants. Variants include T3D^PL^ and mutants with alterations in the sialic acid-binding domain and with the added RGD motif. Data represent mean ± SD (*n* = 3–4). Statistical significance was determined by the one-way ANOVA with Dunnett’s multiple comparisons test in GraphPad Prism v10.4; no significant differences were observed in average σ1 trimer levels (*P* > 0.05).

Given that T3D^PL^ exhibits high average σ1 trimers per virion, it was unlikely that mutations in the sialic acid-binding domain increased binding at physiological temperature by elevating σ1 trimers per virion. However, the possibility needed to be tested empirically. The levels of σ1 trimers per virion were assessed by agarose electrophoresis (Fig. 8A). The intensities for each of the 13 bands were quantified as a percentage of the total for a given virus (Fig. 8B), and the average number of σ1 trimers per particle was calculated (Fig. 8C). As previously found, T3D^PL^ particles averaged 9.2 ± 0.4 σ1 trimers per virion. Variants with truncated σ1 also showed 13 bands, although all bands representing 1–12 trimers migrated faster due to the lower molecular weight of the truncated σ1 protein (Fig. 8A). Importantly, the band representing zero trimers co-migrated across all samples, confirming the structural integrity of the viral capsids. No significant differences were found in the average number of σ1 trimers per virion among the variants (Fig. 8C), indicating that the increased attachment of mutants with sialic acid-binding domain changes or RGD domain addition was not due to a higher density of attachment proteins, but rather the enhanced thermal stability of the individual σ1 interactions at 37°C.

### Mutations in the sialic acid-binding domain enhance sialic acid-mediated binding at physiological temperature (37°C)

**Fig 9:**
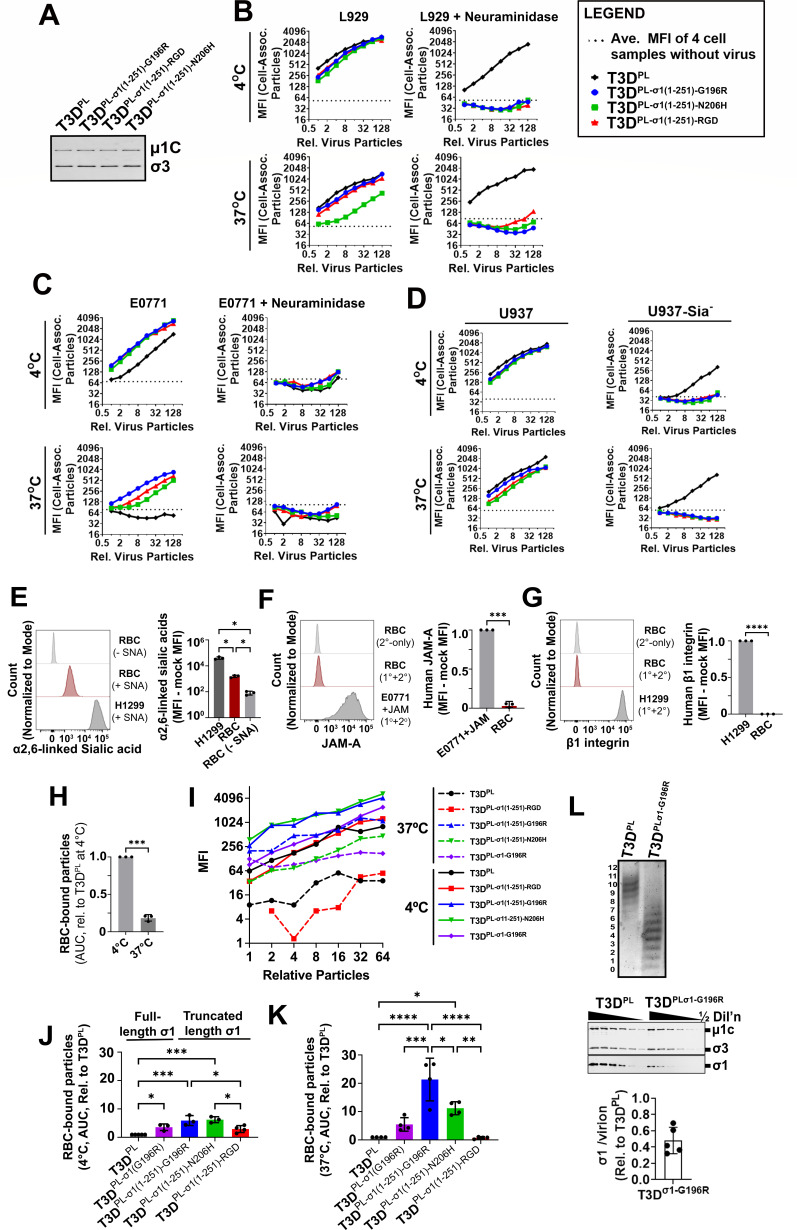
Mutations in the sialic acid-binding domain permit high-affinity, receptor-independent attachment by increasing binding to sialic acids at physiological temperature (37°C). (**A**) Representative western blot analysis of outer capsid proteins µ1C and σ3, detected using anti-reovirus polyclonal serum. (**B and C**) L929 (**B**) and E0771 (**C**) cells were treated with PBS or neuraminidase (+neuraminidase) for 1 h at 37°C to deplete cell surface sialic acids. Viruses indicated in the legend were incubated with cells for 1 h at 4°C or 37°C in the presence of NH_4_Cl, washed, and cells processed for flow cytometric analysis using σ3-specific antibodies. MFI reflects the level of cell-associated virus particles. (**D**) Virus-cell association was measured as in panel **C**, without neuraminidase treatment, using parental U937 cells or U937 cells deficient in sialic acids (U937-Sia^-^). (**E**) Flow cytometric detection of α2,6-linked sialic acids on RBCs using fluorescently labeled SNA lectin. Representative histograms (left) show unstained RBCs (light gray), SNA-stained RBCs (red), and SNA-stained H1299 cells (dark gray). *n* = 3. (**F**) Flow cytometric detection of JAM-A on RBCs using a primary/secondary antibody system specific for hJAM-A. Representative histograms (left) show RBCs with secondary antibody only (light gray), primary/secondary-stained RBCs (red), and E0771+JAM cells (dark gray). *n* = 3. (**G**) Flow cytometric detection of β1 integrins on RBCs using primary/secondary antibodies specific for human β1 integrin. Representative histograms (left) show RBCs with secondary antibody only (light gray), primary/secondary-stained RBCs (red), and H1299 cells (dark gray). *n* = 3. (**H**) RBCs were incubated with particle-normalized T3D^PL^ at serial dilutions starting at 1.9 × 10^5^ particles for 1 h at 4°C or 37°C. Unbound virions were removed by PBS washes prior to fixation and immunostaining for outer capsid proteins, followed by flow cytometric analysis. *n* = 3. (**I–K**) RBCs were incubated with particle-normalized T3D^PL^ or variant viruses at serial dilutions starting at 1.9 × 10^5^ particles for 1 h at 4°C (**I and J**) or 37°C (**I and K**). Following removal of unbound virions by PBS washes, cells were fixed, immunostained for outer capsid proteins, and analyzed by flow cytometry. Absolute MFI values (**I**) were used to calculate the AUC for each virus across all independent experiments, normalized to T3D^PL^ at the corresponding temperature for each independent experiment (*n* = 3–5). (**L**) Levels of σ1 per virion for full-length T3D^PLσ1-G196R^ were assessed by agarose gel electrophoresis (top) and quantitative serial dilution-based western blot analysis using anti-σ3 and anti-µ1 monoclonal antibodies and anti-σ1 tail polyclonal antibodies (middle). Bottom: relative average σ1 per virion calculated relative to T3D^PL^ from five independent virus preparations based on σ1 to (σ3 + µ1) protein ratios determined by western blot analysis. Data represent mean ± SD. Statistical significance was determined using the one-way ANOVA with Tukey’s multiple comparisons test (**E, J, and K**) or the paired *t*-test (**F, G, and H**) in GraphPad Prism v10.4. (ns = *P* > 0.05; *P < 0.05; **P < 0.005; ***P < 0.001; and ****P < 0.0001).

To determine whether σ1-truncation variants associate with cells via sialic acid-dependent mechanisms, virus particles were equalized (Fig. 9A), and attachment to L929 cells (Fig. 9B) or E0771 cells (Fig. 9C) was assessed following neuraminidase treatment or mock treatment with PBS alone. Neuraminidase treatment eliminated attachment of T3D^PL-σ1(1-251)-G196R^, T3D^PL-σ1(1-251)-N206H^, and T3D^PL-σ1(1-251)-RGD^ to L929 cells (Fig. 9B) and E0771 cells (Fig. 9C) at both 4°C and 37°C, indicating that attachment was sialic acid dependent in all cases. The attachment of T3D^PL^ to E0771 cells at 4°C was also sialic acid dependent and was eliminated when E0771 cells were treated with neuraminidase (Fig. 9C). T3D^PL^ could, however, attach to neuraminidase-treated L929 cells since they provide JAM-A for attachment through the σ1 head domain of T3D^PL^ (Fig. 9B). As a complementary, enzyme-independent approach, attachment of σ1-truncation variants was evaluated on U937 pro-monocytic cells with or without genetic knockout of key sialic acid biosynthesis enzymes (77). Unlike T3D^PL^, which attached to both wild-type U937 cells and sialic acid-depleted U937 cells (U937-Sia^-^), T3D^PL-σ1(1-251)-G196R^, T3D^PL-σ1(1-251)-N206H^, and T3D^PL-σ1(1-251)-RGD^ attached to wild-type U937 cells but failed to attach to U937-Sia^-^ cells at 4°C and 37°C, confirming that these σ1-truncation variants depend on sialic acids for attachment (Fig. 9D).

Finally, to determine whether sialic acids are not only necessary but also sufficient for attachment by the σ1-truncation variants, RBCs were chosen as a model system, as they express abundant sialic acids (Fig. 9E) but limited JAM-A (Fig. 9F) and β1 integrin (Fig. 9G). Moreover, RBCs offer a fourth complementary approach to study attachment at 37°C without the need for CPZ, NH_4_Cl, or fixation since they do not internalize virus. RBCs were incubated with serial dilutions of equivalent particle numbers of T3D^PL-σ1(1-251)-G196R^, T3D^PL-σ1(1-251)-N206H^, and T3D^PL-σ1(1-251)-RGD^, alongside T3D^PL^ for 1 h at either 4°C or 37°C. Following flow cytometry to quantify RBC-bound virus particles, AUC was calculated as in Fig. 6 and 7 as a measure of relative cell attachment. Similar to E0771 cells (Fig. 6A and C), T3D^PL^ displayed reduced attachment at 37°C compared to 4°C on RBCs (*P* = 0.0008; Fig. 9H). Because subsequent experiments at 4°C and 37°C with additional viruses were conducted separately to minimize processing time, the AUC for binding at each temperature was then calculated relative to T3D^PL^ at the given temperature. Compared to T3D^PL^, the G196R and N206H σ1-truncation variants showed improved sialic acid-dependent attachment to RBCs at 4°C (Fig. 9I and J: 5.9 ± 1.8-fold, *P* = 0.0003; and 6.3 ± 1.1-fold, *P* = 0.0001, respectively), and a more pronounced improvement at 37°C (Fig. 9I, K: and 21.3 ± 7.5-fold, *P* < 0.0001; and 11.2 ± 2.3, *P* = 0.01, respectively). In contrast, T3D^PL-σ1(1-251)-RGD^ did not exhibit a significant increase in RBC attachment relative to T3D^PL^ at 37°C (0.8 ± 0.4-fold, *P* > 0.99) or 4°C (2.9 ± 1.2-fold, *P* = 0.12), consistent with its design to target integrins rather than carbohydrates. Notably, the failure of the RGD-truncated variant to bind RBCs, despite its high infectivity on E0771 cells, confirms that truncation alone is insufficient to improve sialic acid-dependent attachment; rather, specific mutations like G196R are required to stabilize the interaction at physiological temperatures.

A G196R mutation was previously identified in the full-length σ1 protein of reovirus variants capable of infecting JAM-A-deficient U118MG cells (17); therefore, we also generated full-length σ1 containing the G196R mutation (T3D^PL-σ1(G196R)^) for comparison of attachment to RBCs (Fig. 9I through K). T3D^PL-σ1(G196R)^ showed enhanced RBC attachment relative to T3D^PL^ at 4°C (3.6 ± 1.2-fold, *P* = 0.04). At 37°C, T3D^PL-σ1(G196R)^ had a non-significant trend toward increased RBC association relative to T3D^PL^ (5.5 ± 2.4-fold, *P* = 0.45). Levels of σ1 per virion were drastically lower for T3D^PL-σ1(G196R)^ relative to T3D^PL^ (Fig. 9L) and the truncated σ1 variants (Fig. 8), likely accounting for the decreased attachment of the G196R mutant in the context of full-length relative to truncated σ1.

Together, these results demonstrate that G196R and N206H mutations directly enhance sialic acid-dependent attachment by conferring thermal stability to the virus-cell interaction at physiological temperatures. While the σ1-truncated RGD variant serves as an essential proof-of-concept that reovirus can be engineered to utilize alternative receptors like integrins, it is the spontaneous mutations within the sialic acid-binding domain that reveal the natural adaptive strategy of the virus. Under the selective pressure of insufficient primary receptor attachment, mutations in the sialic acid-binding pocket achieve 37°C stability, effectively bypassing the requirement for the primary receptor.

## DISCUSSION

**Fig 10:**
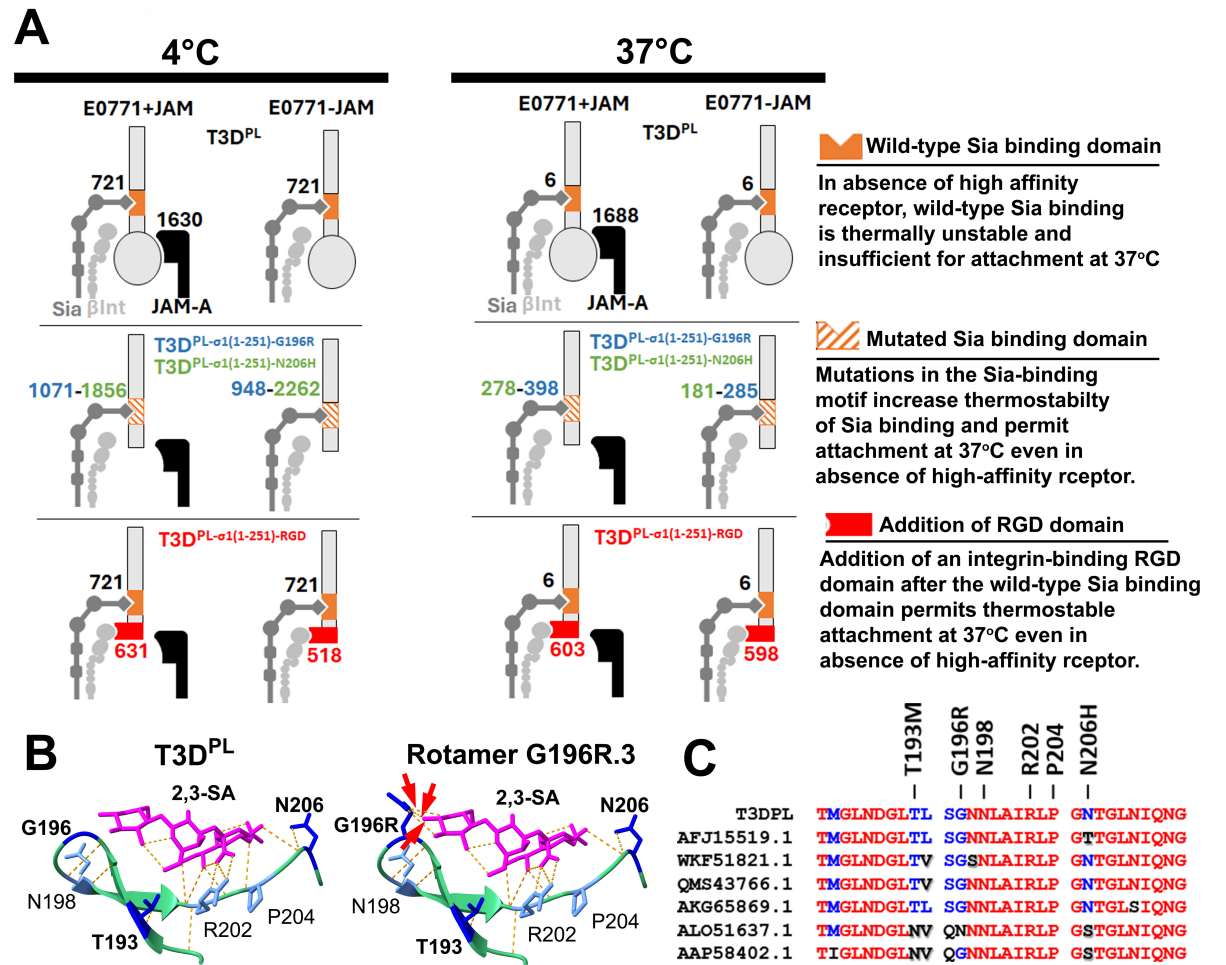
Mutational enhancement of σ1-mediated receptor binding and structural basis of sialic acid interaction. (**A**) Model depiction of relative binding strengths deduced from experimental mean AUCs between the sialic acid-binding domain (orange) to sialic acids (dark gray, Sia), the JAM-A-binding domain (circular head of σ1) to JAM-A (black), and the RGD domain (red) to β-integrin (light gray, βInt). Where “~” is indicated, relative binding strength was deduced by subtracting the total binding measured in the JAM-A-deficient condition from the domain-specific binding strength. (**B**) Structural models of the T3D^PL^ σ1 body domain were generated using UCSF ChimeraX (v1.9). Wild-type (bordered) and mutant σ1 structures were created by introducing identified substitutions. Predicted hydrogen bonds with α2,3-linked sialic acid (PDB: 3S6X) were assessed using default cutoffs; a representative G196R rotamer shows novel hydrogen bonds between the arg196 and sialic acid (red arrow). (**C**) Amino acid sequence alignment of the σ1 body domain encompassing the sialic acid-binding pocket (NCBI). Mutations identified through passage (G196R, T193M, and N206H) are annotated alongside known sialic acid-binding residues (N198, R202, and P204).

Reovirus Type 3 Dearing (T3D^PL^) is a safe, versatile model for viral biology and a promising oncolytic candidate, yet patient responses in breast cancer trials remain variable. The findings demonstrate that the poor susceptibility of E0771 breast cancer cells to T3D^PL^ is a direct consequence of high-affinity receptor deficiency, which can be fully reversed by exogenous JAM-A expression (Fig. 4). Consequently, E0771 cells provide a valuable syngeneic model for studying JAM-A-deficient breast cancers. Second, the studies reveal that T3D^PL^ attachment to JAM-A-deficient E0771 cells and RBCs is thermally unstable, leading to an approximately 100-fold reduction in binding at physiological temperature (37°C; Fig. 6, 7 and 9). While most attachment studies are performed at low temperatures, these results highlight the necessity of using physiological temperatures to accurately predict viral tropism and therapeutic efficacy. Third, σ1-truncation variants bearing single amino acid mutations (G196R, N206H/K/Y, and T193M) or the RGD domain successfully bypass high-affinity receptor deficiency to achieve robust infection at 37°C. Mechanistically, the σ1 mutations directly enhance sialic acid-dependent binding thermostability (Fig. 9). Notably, the G196R mutation conferred greater benefit within a truncated σ1 protein than in the full-length protein, because the G196R mutation reduced levels of σ1 on virions in the context of the full-length protein. Altogether, these results suggest that high-affinity receptor deficiency in breast cancer can be compensated for via simple genetic modifications to σ1 (Fig. 10A), potentially broadening the therapeutic window for reovirus. Finally, the rapid adaptation of reovirus underscores the evolutionary flexibility of sialic acid-binding domains and provides a robust model for studying viral receptor adaptation.

### Biological necessity of stabilizing mutations for JAM-A-independent attachment

A potential limitation of this study is the absence of a σ1-truncation variant possessing a purely WT sialic acid-binding domain as a baseline control. However, our results suggest that such a variant is biologically non-viable under standard propagation conditions. Despite repeated reverse genetics attempts, the virus consistently incurred compensatory mutations in the sialic acid-binding pocket (e.g., T193M, G196R, and N206K) or reverted to a full-length phenotype by eliminating the stop codon. This consistent selective pressure suggests that the WT sialic acid-binding domain lacks the requisite thermal stability to support propagation at 37°C without a high-affinity head domain. This deficiency is further validated by internal controls: the σ1 -RGD variant, which retains a WT sialic acid-binding domain, behaves identically to T3D^PL^ on RBCs, and fails to show enhanced glycan-mediated attachment at 37°C. Furthermore, the comparable attachment of T3D^PL^ and the truncation variants at 4°C confirms that the WT tail is functional but specifically restricted at physiological temperatures. Collectively, these data demonstrate that JAM-A-independent infection requires specific, stabilizing modifications to the sialic acid-binding domain rather than being a mere consequence of σ1 truncation.

### Clinical relevance of receptor status in breast cancer therapy

Elevated JAM-A protein and RNA levels correlate with poor prognosis for several cancers, including breast cancer (27–32). Furthermore, inhibiting JAM-A via knockdown, monoclonal antibodies, or antagonistic peptides significantly reduces tumor volume in xenograft models (28, 29, 31, 78). These findings highlight the therapeutic potential of reovirus, since aggressive, high-prognosis breast cancers likely express sufficient JAM-A to facilitate viral oncolysis. However, because the majority of breast cancers are JAM-A-low or JAM-A-negative, T3D^PL^ efficacy may be severely limited by receptor availability in most clinical cases, underscoring the potential benefit of engineered variants that bypass JAM-A dependency. Ultimately, strategies to improve targeting of JAM-A-deficient breast cancers must be validated in syngeneic mouse models to determine whether enhanced *in vitro* infectivity translates to superior *in vivo* anti-tumor efficacy and immune activation.

### Distinct molecular determinants of binding strength and thermostability

The results clarify the relative contributions of binding strength and thermostability for interactions involving JAM-A, sialic acid, and β1 integrin (Fig. 10A). Specifically, JAM-A engagement is both strong and thermally stable, whereas the wild-type sialic acid-binding domain, despite possessing ~50% of JAM-A binding strength at 4°C, is highly thermolabile. In contrast, the G196R/N206H mutations achieve ~100% of JAM-A binding strength at 4°C and exhibit ~24-fold higher thermostability than the wild-type σ1. These data support the conclusion that binding strength and thermostability are governed by distinct molecular determinants. For example, the moderate-strength RGD motif and the wild-type sialic acid-binding domain exhibit comparable affinities at 4°C, yet only the RGD interaction endures at physiological temperatures. Similarly, the G196R, T193M, and N206H mutations, despite displaying strong binding, remain moderately thermosensitive. These observations suggest that even strong, surface-level interactions may be highly susceptible to dissociation at physiological temperature. In contrast, the mid-strength RGD and high-strength JAM-A interactions resist these biophysical stresses, indicating a more robust and structurally stable engagement mechanism. These principles likely extend to other viruses that utilize sialic acids, such as adenovirus, rotavirus, and SARS-CoV-2 (51, 79–88). Comparing these to viruses that rely on sialic acids as primary receptors, such as influenza A and coxsackievirus A24, could further elucidate how glycan-binding domain features influence the thermal stability of viral attachment.

### Structural basis and modeling of enhanced sialic acid engagement

Previous studies found that residues Asn198, Arg202, and Pro204 are essential for sialic acid engagement, forming stabilizing hydrogen bonds and van der Waals contacts with sialic acid (51). The G196R, N206H/Y/K, and T193M mutations flank the three critical residues. To explore potential biochemical means by which the mutations could influence sialic acid attachment, structural modeling analysis was performed using existing crystallography structures of the σ1 tail associated with sialic acids (23). Substitution rotamers were evaluated to identify potential novel interactions with α2,3-linked and α2,6-linked sialic acids or the σ1 backbone. Multiple G196R rotamers were predicted to form novel hydrogen bonds directly with both α2,3- and α2,6-linked sialic acids, likely accounting for increased stability of interaction (Fig. 10B). In contrast, T193M and N206H/Y/K mutations did not form new direct bonds with the carbohydrate; their enhanced binding therefore may stem from new intramolecular hydrogen bonds with the σ1 backbone or stabilized water/ion bridges to the glycan. Alternatively, these mutations could stabilize favorable σ1 conformations that optimize sialic acid trapping or promote binding to different glycan backbones similar to the receptor-switching phenomena observed for avian influenza viruses (89–92). While our modeling provides valuable hypotheses, definitive biochemical conclusions will require future high-resolution structural studies, such as cryo-EM or X-ray crystallography, to elucidate how these mutations affect thermal stability of the σ1-sialic acid interactions.

### Receptor compensation and the evolutionary trade-off of virus attachment strength

Finally, the analysis of σ1 mutants provides a powerful example of evolutionary selection pressure driving a “receptor compensation” mechanism. Experimental removal of the primary, high-affinity receptor binding domain favored the selection of mutations (e.g., G196R, N206H/Y/K, and T193M) that significantly increased the attachment strength and stability of the secondary, typically longer-strength sialic acid interaction. This finding illustrates a fundamental principle: when confronted with receptor-limited environments, certain viruses can rapidly shift receptor usage to alter tropism. Notably, the RGD-engineered variants were consistently sequenced and did not acquire adaptive mutations in the sialic acid-binding pocket, suggesting that RGD-driven attachment provided sufficient compensation to remove the selection pressure for enhanced sialic acid attachment. To determine if G196R, N206H/Y/K, and T193M mutations in the sialic acid-binding domain also occur naturally, we analyzed 43 sequenced serotype 3 σ1 isolates from the NCBI database, focusing on the 30 amino acids surrounding the key sialic acid binding residues N198, R202, and P204 (66). While the majority of sialic acid-binding regions were identical to T3D^PL^, several environmentally derived reoviruses exhibited substitutions at positions 193, 196, and 206 (Fig. 10C). Though none harbored the exact mutations identified in our study, these polymorphisms may similarly influence the efficiency of sialic acid engagement. Notably, however, the facts that the sequence of the T3D^PL^ sialic acid-binding region is representative of the majority of sequenced serotype 3 reovirus σ1 proteins, that reoviruses are successfully widespread among mammals (93–97), and that T3D^PL^ represents a “mediocre” rather than “strong” sialic acid attachment phenotype may suggest that sub-maximal cell attachment comes with advantages. Supporting this idea, sub-maximal cell attachment can increase the distance of reovirus spread among tumor cells (35), and neuraminidase activity is essential for detachment of influenza and dissemination (98). Thus, moderate sialic acid-binding may confer advantages, such as facilitating viral release and localized spread. Similar shifts toward optimal, rather than maximal, glycan engagement have been suggested for other viruses, including SARS-CoV-2 (99–118). Together, these findings position reovirus as a safe and tractable model for investigating how viruses fine-tune receptor engagement under selective pressure.

## MATERIALS AND METHODS

### Cell lines

All cell lines were cultured at 37°C in a humidified incubator with 5% CO_2_ and maintained in medium supplemented with 5%–15% fetal bovine serum (FBS; Invitrogen), 1× non-essential amino acids (Sigma), and 1 mM sodium pyruvate (Sigma). L929 and HEK 293T/17 cells (ATCC) were maintained in Minimal Essential Medium (MEM; Sigma) containing 5% FBS and Dulbecco’s Modified Eagle’s Medium (DMEM; Sigma) supplemented with 10% FBS, respectively. TUBO cells, provided by Dr. Lorena Landuzzi (Istituto Ortopedico Rizzoli), and BHK-T7 cells, gifted by Dr. Ursula Buchholz (National Institutes of Health), were cultured in DMEM containing 10% FBS; BHK-T7 cells were additionally supplemented with 1 mg/mL G418 sulfate every second passage. EMT6 (CRL-2755) and E0771 cells (CRL-3461), kindly provided by Dr. Mary Hitt (University of Alberta), were maintained in DMEM/F12 (Invitrogen) containing 15% FBS and Roswell Park Memorial Institute 1640 Medium (RPMI; Sigma) supplemented with 10% FBS, respectively. U937 and sialic acid-deficient U937 cells were generously gifted by Dr. Matt Macauley (University of Alberta) and maintained in 10% FBS containing RPMI. All cell lines were routinely tested for mycoplasma contamination by polymerase chain reaction.

### Site-directed mutagenesis

Reovirus strain T3D^PL^, provided by Dr. Patrick Lee (Dalhousie University), was used as the genetic background for all recombinant viruses generated in this study. The Shmulevitz laboratory previously cloned all 10 reovirus genes, including the S1 segment encoding σ1, into plasmids under the control of a T7 promoter (66). Mutations, including an early stop codon or an RGD peptide insertion, were introduced into the S1 gene by PCR using iPROOF DNA polymerase (BioRad) with 50 ng of template DNA and 125 ng of each primer (early stop codon: forward 5′-GTAGCGTTCATAGGTTATCAACCGGCGTTTTCTTCCAC-3′, reverse 5′-GTGGAAGAAAACCCCGGTTGATAACCTATGAACGCTAC-3′; RGD insertion: forward 5′-GGATAGGCGCACTTGAGCAATGATAAGGCGGTAGTTG-3′, reverse 5′-CAACTACCGCCTTATCATTGCTCAAGTGCGCCTATCC-3′). Following PCR, reactions were held at 10°C and treated with DpnI (Thermo Fisher Scientific) for 2 h at 37°C to digest the parental (methylated) plasmid DNA. Ten nanogram of each mutated plasmid was then transformed into electrocompetent Top10 *Escherichia coli* cells using a 2.5 mm cuvette at 2.5 kV. Transformed cells were recovered in SOC medium (20 g/L tryptone, 5 g/L yeast extract, 0.5 g/L NaCl, and 20 mM glucose) for 1 h at 37°C, plated on LB agar containing ampicillin, and incubated at 37°C for 16 h. Single colonies were cultured in LB broth with 100 µg/mL ampicillin. Plasmids were purified using the GeneJET Plasmid Miniprep Kit and sequenced to confirm the desired mutations.

### Reverse genetics

Recombinant reoviruses were rescued using a plasmid-based reverse genetics system (119, 120). Briefly, 10 plasmids encoding the complete T3D^PL^ genome (with or without S1 modifications) were co-transfected into BHK-T7 cells, which stably express T7 RNA polymerase. To enhance virus recovery, a plasmid encoding the C3P3 fusion protein was included. Cells were transfected with a total of 2.25 µg of plasmid DNA in 40 µL Opti-MEM reduced serum medium (Invitrogen) using TransIT-LT1 transfection reagent (Mirus) at a 3:1 reagent to DNA ratio. The medium was replaced 16 h post-transfection, and virus-containing lysates were harvested 5 days post-transfection by scraping cells into suspension, followed by a series of three freeze-thaw cycles.

### Virus purification

Viruses were propagated in suspension L929 cell cultures from seed stock lysates to maintain genetic identity, extracted twice from infected cells with Vertrel XF (Dupont), and purified by cesium chloride gradient ultracentrifugation as described previously (121). Purified virus was dialyzed in virus dilution buffer (150 mM NaCl, 10 mM MgCl_2_, and 10 mM Tris pH 7.4) using dialysis tubing with a 12,000–14,000 Da molecular weight cut-off, with three buffer exchanges. The dialyzed virus was then collected and stored at 4°C until use.

### Virus titration

Virus titers were determined by plaque assay on L929 cells. Virus-infected cell lysates were serially diluted in serum-free growth medium and adsorbed onto confluent L929 monolayers for 1 h at 37°C with 5% CO_2_, with manual rocking every 10 min. Following infection, cells were overlaid with a molten mixture of 2% (wt/vol) agar, 2× Joklik’s modified essential medium (pH 7.4; Sigma), and 1× MEM at a 1:1:2 volume ratio. Overlays were allowed to solidify at room temperature for 30 min before plates were returned to the incubator. Plates were incubated undisturbed until plaques were visible. Cells were fixed with 4% PFA in 1× PBS for 30 min at room temperature, after which the agar overlays were carefully removed. Monolayers were further fixed with methanol for 10 min at room temperature, then stained with 1% (wt/vol) crystal violet. Excess stain was removed, wells were rinsed with water, and plates were air dried at room temperature for 24 h. Plaques were imaged using a ChemiDoc MP Imaging System (BioRad), and virus titers were calculated.

### Virus particle normalization

All viruses were CsCl gradient purified; therefore, the particle numbers of genome-containing viruses were deduced from optical density at 260 (1 unit at OD_260_ equivalent to 2.1 × 10^12^ particles/mL) (122). For precise equalization of particles, five twofold dilutions of virus particles were subjected to SDS-PAGE alongside T3D^PL^, stained with Coomassie, and relative particles to T3D^PL^ were calculated from standard curve analysis. For each binding experiment, input virions were subjected to SDS-PAGE and western blot analysis for reovirus outer capsid proteins (rabbit anti-reovirus polyclonal antibody; gift from Dr. Patrick Lee [Dalhousie University]) to confirm equivalent particles between viruses in each experiment.

### Flow cytometry and cell staining

Flow cytometry was used to quantify virus attachment, intracellular viral replication, and cell-surface receptor expression. Cells were collected using Cell Stripper (Corning), and a total of 2 × 10^5^ cells were fixed with 4% PFA for 30 min at room temperature (or 4°C/37°C for virus attachment). Cells were then washed three times with flow buffer (PBS supplemented with 3% FBS and 1 µM EDTA) at 350 × *g* for 5 min. Where intracellular staining was required, cells were permeabilized using 0.1% Triton X-100 in flow buffer. Cells were stained with a monoclonal anti-σ3 antibody (clone 10G10, DSHB) or a monoclonal anti-σNS antibody (clone 2A9, DSHB) to detect cell-bound reovirus particles or intracellular reovirus replication, respectively. To quantify cell-surface receptor expression, cells were incubated with monoclonal antibodies specific for murine JAM-A (clone BV11, Millipore Sigma), murine β1 integrin (eBioscience), human JAM-A (CSTEM27, Thermo Fisher Scientific), or human β1 integrin (clone P5D2, DSHB). Fluorescent labeling was performed using Cy5-conjugated secondary antibodies. All antibody incubations were carried out at room temperature for 30 min in the dark, followed by three washes with PBS. For quantification of α2,6-linked sialic acids, 2 × 10⁵ cells were stained with fluorescently labeled SNA lectin (Vector Labs) for 20 min, fixed with 4% PFA, and washed as described above. Fluorescence was acquired on a LSRFortessa flow cytometer (BD Biosciences) at the University of Alberta Flow Cytometry Facility, with a minimum of 10,000 events recorded per sample. Data analysis was performed using FlowJo v10 (BD Biosciences).

### Western blot analysis

Protein samples were diluted in 5× protein sample buffer (250 mM Tris-HCl pH 6.8, 5% SDS, 45% glycerol, 9% β-mercaptoethanol, and 0.25% bromophenol blue) to a final concentration of 1× and denatured by boiling at 100°C for 10 min. Proteins were resolved by 10% SDS-PAGE at 100V and transferred onto nitrocellulose membranes using the Trans-Blot Turbo Transfer System (Bio-Rad) under semi-dry conditions (1.0A, up to 25V, for 30 min). Membranes were blocked for 1 h at room temperature in 3% newborn calf serum (NBCS) prepared in TBS-T (0.1% Tween-20). After blocking, membranes were incubated for 1 h at room temperature with one of the following primary antibodies: mouse anti-β-actin monoclonal antibody (clone 8H10D10, Cell Signaling Technology); mouse anti-FLAG IgG monoclonal antibody (clone M2, Millipore Sigma); rabbit anti-reovirus polyclonal antibody; or rabbit anti-σ1N monoclonal antibody (gifts from Dr. Patrick Lee [Dalhousie University]). Membranes were washed three times in TBS-T and incubated with HRP-conjugated secondary antibodies. After three additional washes in TBS-T, membranes were developed using ECL Plus Western Blotting Substrate (Thermo Fisher Scientific). Chemiluminescent signals were detected using a ChemiDoc MP Imaging System (BioRad). Densitometric analysis was performed with ImageQuant TL software (GE Healthcare Life Sciences), and final figures were prepared using Adobe Photoshop.

### Reovirus infectivity

Cells were seeded into 24-well plates (2 cm^2^/well) and exposed to virus at 90% confluency for 1 h in a 50 µL infection volume, with plates rocked gently every 5–10 min. Following the initial infection, complete medium was added, and cells were incubated for an additional 18 h at 37°C. For flow cytometric analysis, cells were collected after 18 h of infection by incubation with CellStripper for 10 min at room temperature and fixed with 4% PFA at room temperature for 30 min. Samples were then processed for flow cytometric analysis as described previously.

For immunofluorescence microscopy, cells were washed once with PBS and fixed with room temperature 4% PFA for 30 min at room temperature. Fixed cells were washed three times with PBS and blocked for 1 h in 3% NBCS prepared in PBS with 0.1% Triton X-100. Cells were then incubated with monoclonal anti-σNS antibodies (clone 2A9, DSHB) for 1 h at room temperature, washed three times with PBS, and incubated with Cy5-conjugated secondary anti-mouse antibodies for 1 h each at room temperature. After three additional washes, nuclei were stained with DAPI (Invitrogen) for 10 min in PBS. Fluorescence was visualized on an EVOS FL Auto Imaging System (Life Technologies), and images were assembled using Adobe Photoshop.

### Attachment and detachment of reovirus particles

To assess reovirus attachment and detachment in the presence of CPZ, cells were seeded into 24-well plates (2 cm^2^/well) and cultured to confluency. Cells were then pre-incubated with or without 22 µg/mL of CPZ in serum-containing medium at 37°C or 4°C. Pre-treatment was 3 h for Fig. 2 and 6A; otherwise, a 1 h pre-treatment was used, which was sufficient to maintain surface-associated virions during the 1 h incubations at 37°C. After the pre-incubation, cells were exposed to virus particles as indicated for 1 h at either 4°C or 37°C. Supernatants were collected, and cells were washed three times with temperature-matched PBS to remove unbound virions, or cells were incubated in fresh serum-free medium for an additional hour at 37°C (followed by three washes with temperature-matched PBS) before collection and fixation. For flow cytometry analysis, cells were detached using CellStripper and fixed with temperature-matched 4% PFA for 30 min. For western blot analysis, cells were lysed directly in RIPA buffer (50 mM Tris-HCl pH 7.4, 150 mM NaCl, 1% IGEPAL CA-630 [NP-40], and 0.5% sodium deoxycholate) supplemented with protease inhibitor cocktail and processed as described previously.

For measuring cell-associated particles on cells under conditions of NH_4_Cl and ghosts, cells were used at 1 × 10^6^ cells per condition, mixed with twofold serial dilutions of virus particles starting at 1 × 10^10^ particles per condition (10,000 particles/cell). For attachment with NH_4_Cl, untreated cells were subjected to attachment of virus and subsequent analysis in the presence of 10 mM NH_4_Cl prior to fixation. For attachment with cell ghosts, cells were fixed in 4% PFA for 20 min on ice, washed with PBS, and then subjected to virus attachment at respective temperatures without any treatments. For neuraminidase treatment, cells were treated with neuraminidase from *Clostridium perfringens* (200 mU/1 × 10^7^ cells; Sigma) for 1 h at 37°C, washed with PBS, and subjected to virus attachment at respective temperatures.

### Retroviral vector production and stable cell line generation

Recombinant retroviruses were generated to stably express transgenes in mammalian cells via random genomic integration. The open reading frame (ORF) of human JAM-A (Plasmid #70,073, Addgene) was subcloned into the pBABEpuro vector (Plasmid #21,836, Addgene) using BamHI and SalI restriction digestion followed by ligation with 1U/µL T4 DNA ligase (Invitrogen) for 1 h at room temperature. Ligated products were transformed into competent Top10 *E. coli* cells using a 30 min incubation on ice, a 45 s heat shock at 42°C, and a 1 h recovery in SOC medium at 37°C, before plating on LB agar containing 100 mg/mL ampicillin. Positive clones were confirmed by restriction digest and Sanger sequencing.

Retroviral particles were produced by co-transfecting HEK 293T/17 cells in a six-well plate (10 cm^2^) at ~70% confluency with 1,720 ng pBABE:ORF, 1,150 ng pHIT60 encoding gag-pol (kindly provided by Dr. Louis Staudt, NCI), and 1,150 ng pMD2.G encoding VSV-G (Plasmid #12,259, Addgene). DNA was mixed with 6.87 µL Lipofectamine 2000 transfection reagent in 250 µL of Opti-MEM, then added to cells. Transfection was allowed for 4–6 h before replacing the media. Viral supernatants were collected at multiple timepoints between 20 and 52 h post-transfection, pooled, filtered (0.45 µm), and either used immediately or stored in aliquots at −80°C. E0771 cells were transduced the following day with serial dilutions of retrovirus in the presence of 8 µg/mL Polybrene (Thermo Fisher Scientific). After a 3 h incubation at 37°C, viral supernatants were replaced with fresh media, and cells were selected with 2 µg/mL puromycin for 48 h. Surviving cells were expanded under puromycin selection, and stable transgene expression was confirmed by flow cytometry.

### RBC processing and reovirus attachment

Human type B RBCs, collected under approved protocols and provided with informed consent, were processed to generate viable suspensions for virus binding assays. Whole blood was collected into EDTA-coated tubes to prevent coagulation and stored on ice until processing. Samples were centrifuged at 2,000 × *g* for 10 min at 4°C to separate plasma from cellular components. The plasma supernatant was carefully removed, and the RBC pellet was resuspended in PBS. This washing step was repeated twice, each time centrifuging at 2,000 × *g* for 5 min at 4°C to remove residual plasma proteins and leukocytes. After the final wash, RBCs were resuspended in PBS to a final concentration of 50% (vol/vol). RBC suspensions were stored at 4°C and used within 2 weeks of collection to maintain viability and surface glycan integrity critical for virus-receptor interaction studies.

To assess sialic acid-dependent attachment, 8 × 10^5^ RBCs were aliquoted into FACS tubes and incubated at either 4°C or 37°C, as indicated. Twofold serial dilutions of virus, starting at 1.9 × 10^5^ particles, were incubated with the RBCs for 30 min with gentle mixing every 10 min to prevent sedimentation. Following incubation, the unbound virus was removed by washing with temperature-adjusted flow buffer at 350 × *g* for 5 min. Bound virus particles were then fixed with temperature-adjusted 4% PFA for 30 min, followed by an additional wash under the same conditions. Samples were processed for flow cytometric analysis as described previously, and the AUC was calculated using GraphPad Prism (v10.4).

### Structural modeling of the reovirus sialic acid-binding pocket

To investigate how point mutations within the σ1 attachment protein affect sialic acid interactions, structural modeling of the reovirus σ1 sialic acid-binding pocket was performed using UCSF ChimeraX (v1.9). The images represent the σ1 body domain of T3D^PL^. Wild-type and mutant σ1 structures, including T193M, G196R, and N206H/Y/K, were generated by introducing amino acid substitutions using the Rotamers and Swapaa tools in ChimeraX. Hydrogen bonding interactions with α2,3-linked (PDB ID: 3S6X) and α2,6-linked (PDB ID: 3S6Y) sialic acids were predicted using the FindHBond tool with default distance and angle cutoffs.

## Data Availability

No new data were generated or analyzed in this study. Data sharing is not applicable to this article.
